# Therapeutic effects of mesenchymal stem cells-derived extracellular vesicles’ miRNAs on retinal regeneration: a review

**DOI:** 10.1186/s13287-021-02588-z

**Published:** 2021-10-07

**Authors:** Ali Rajool Dezfuly, Azadeh Safaee, Hossein Salehi

**Affiliations:** Department of Anatomical and Molecular Biology Sciences, School of Medicine, Isfahan University of Medical Sciences, Isfahan, Iran

**Keywords:** Extracellular vesicles, Retina, miRNA, Mesenchymal stem cells

## Abstract

Extracellular vesicles (EVs), which consist of microvesicles and exosomes, are secreted from all cells to transform vital information in the form of lipids, proteins, mRNAs and small RNAs such as microRNAs (miRNAs). Many studies demonstrated that EVs’ miRNAs have effects on target cells. Numerous people suffer from the blindness caused by retinal degenerations. The death of retinal neurons is irreversible and creates permanent damage to the retina. In the absence of acceptable cures for retinal degenerative diseases, stem cells and their paracrine agents including EVs have become a promising therapeutic approach. Several studies showed that the therapeutic effects of stem cells are due to the miRNAs of their EVs. Considering the effects of microRNAs in retinal cells development and function and studies which provide the possible roles of mesenchymal stem cells-derived EVs miRNA content on retinal diseases, we focused on the similarities between these two groups of miRNAs that could be helpful for promoting new therapeutic techniques for retinal degenerative diseases.

## Introduction

The retina is a part of the central nervous system (CNS) which originates from diencephalon. The inner sensory retina and retinal pigment epithelium (RPE) are two layers of it [1, 2]. The association neurons (amacrine and horizontal cells), the conducting neurons (bipolar and ganglion cells), the photoreceptor neurons (cone and rod receptors), and the supporting Müller cells are four cell groups of inner sensory retina whereas the RPE is made up of cuboidal cells which are organized in one layer[1]. The light photons are transformed to electrochemical signals by the retina and projected to the brain via the optic nerve. The whole process gives the organism the ability of vision [3].

Many people suffer from the blindness caused by retinal degenerations around the world. The death of retinal neurons, same as the CNS, is irreversible and causes permanent damage to the retina. Degenerative inherited retinal diseases such as retinitis pigmentosa and age-related macular degeneration (AMD) are important causes of visual disability [1, 3–6]. The principal reason of retinal degeneration is the loss of photoreceptors, but no effective treatment has been discovered yet [7]. Retina’s structure and anatomical position have made it an ideal tissue for examining new treatment methods such as prosthetic therapy, gene therapy and cell therapy for its neurodegenerative diseases. It is an easily accessible structure of the central nervous system which is quite isolated from the other parts of the body. Researches on cell therapy have become prevalent in recent decades. One of the cell therapy advantages is restricting degeneration via delivering trophic and neuroprotective agents that might inhibit the progression of the visual disease. Another advantage of cell therapy over other methods is the possible differentiation of transplanted cells that might replace the dead cells and restore the function of the tissue [8]. Considering the specifications of stem cells such as their differentiation capacity, multipotency and self-renewal, stem cell therapy has become an important therapeutic approach [1, 3]. Different types of stem cells have been used for retinal differentiation and transplantation including induced pluripotent stem cells (iPSCs), isolated retinal stem cells (RSCs), human embryonic stem cells (hESCs) and mesenchymal stem cells (MSCs) [9, 10]. MSCs do not have the clinical limitations of other stem cells and owing to their immunomodulatory and autologous features, easy isolation and relative abundance, they are more promising choices than other types of stem cells for retinal regeneration [10].

Many studies on regenerative medicine have shown that most of MSCs will be lost in the cell therapy process, this suggests that the main part of tissue regeneration is possibly made by the paracrine factors of the MSCs [11–14]. One of the main components of MSCs paracrine factors which are highly regarded as tissue regenerators are EVs. The inner components of EVs generally consist of proteins and nucleic acids, especially miRNAs [15]. As new studies have suggested that EVs miRNA content seems to play a more important role in retinal regeneration than other components [12], in this review, we will discuss the potential role of MSCs-derived EVs’ (MSC-EVs) miRNAs as a treatment for retinal diseases.

## Mesenchymal stem cells (MSCs)

MSCs are non-hematopoietic stem cells which are derived from various somatic tissues and have the self-renewal capacity. They can be found in different tissues including umbilical cord, embryonic tissues, fetal membranes, dental pulp, adipose tissue, liver, cartilage, skin, breast milk, skeletal muscle, peripheral blood, corneal limbal stroma of the eye and bone marrow [16, 17]. MSCs can migrate to the sites of injury to advance tissue regeneration and suppress the immune reactions by regulating the function of both innate and acquired immune systems [17]. Because of their anti-inflammatory [16], regenerative and immunosuppressive features, they are being used widely in the field of cellular therapy studies nowadays [11]. According to the International Society for Cellular Therapy (ISCT) the minimal requirements of the MSCs are the expression of cell surface markers CD73, CD90 and CD105, and negative expression of CD34, CD45, or CD11b, CD79-α, or CD19, CD14 and HLA-DR markers. The other main requirement is the plastic adherence in standard culture conditions. Moreover, MSCs must be able to differentiate into mesenchymal cells such as chondrocytes, osteoblasts, adipocytes and fibroblasts in vitro [1, 11, 18]. Moreover, researches have shown that MSCs can differentiate into a range of numerous cells such as cardiomyocytes, muscle fibers, renal tubular cells, hepatocytes, pancreatic islands and neurons [11]. So these kinds of cells could be used in many types of tissue regeneration including the retina [12, 16]. For example, Özmert et al. treated 32 patients of retinitis pigmentosa with subtenon space transplantation of Wharton’s jelly mesenchymal stem cells (WJ-MSCs) in a clinical trial. They concluded that the subtenon injection of WJ-MSCs could restrict the disease progression while being completely safe after twelve months of follow-up [19]. Despite the fact that therapeutic use of MSCs was promising, the possible unwanted differentiation of transplanted cells remains a safety issue [20]. Moreover, administration of MSCs for inflammatory bowel disease (IBD) and idiopathic pulmonary fibrosis (IPF) patients who were receiving immunosuppressive drugs shortly before MSC injection caused serious respiratory and gastrointestinal infections, suggesting that applying MSCs in combination or instantly after administering immunosuppressive drugs could be harmful [21].

Also, it has been shown that the positive effects of MSC therapy are substantially due to their trophic and immunosuppressive secreted factors and most of the transplanted cells will not differentiate and integrate into retinal tissue [20, 22]. MSCs secrete various trophic factors including FGF-2, IGF-1, BDNF, HGF, VEGF, IGF1, TGF-β1, bFGF and GDNF which attribute to neuronal survival and regeneration [23].

Recent studies have shown that these kinds of cells also release EVs which play an important part in cellular communications that promote tissue regeneration [11, 24].

## Extracellular vesicles

EVs are secreted vesicles which are approximately found in all body fluids and the extracellular matrix [3]. They are secreted from all cells to transform vital information as lipids, proteins, mRNAs and small RNAs. EVs’ proteins are mostly a representation of their parent cells; however, the number of certain types of molecules such as cytokines, proteinases, chemokines, cell-specific antigens, cytoplasmic enzymes, signal transduction proteins, heat shock proteins and the ones which are related to cell adhesion and membrane trafficking are higher in the vesicles [25]. EVs include exosomes, microvesicles and apoptotic bodies. They are categorized by the proteins which are located on their surface, the range of their size in nanometer, their inner components and their biogenesis pathway [3].

Exosome formation is via the inward budding of the late endosome membranes which are called multivesicular bodies (MVBs). As the MVBs fuse with cell membrane, they would be released in the extracellular space [26]. The size of exosomes is considered as 30–150 nm [3]. Significant physiological and pathological functions have been attributed to exosomes including antigen presentation, inflammation regulation, immunological responses, angiogenesis processes, neuroprotection, regeneration processes, discarding inessential proteins and diffusing pathogens or oncogenes [27]. Exosomes can regulate the cellular status and their features would change in numerous diseases including cancer [28]. This suggests them as diagnostic and therapeutic tools [15]. For example, Galardi et al. showed that proteins that are characteristically associated with retinoblastoma vitreous seeding (RBVS) invasion and metastasis have been upregulated in RBVS exosomes [29]. Exosomes also have a drug delivery function [25, 30]. Schindler et al. demonstrated that exosomes which are loaded with doxorubicin, an anthracycline antibiotic that is prescribed in the treatment process of many kind of cancers, would be absorbed by cells quickly and their inner doxorubicin would be re-distributed from endosomes to the cytoplasm and nucleus of the recipient cells [31].

Another type of EVs that are formed through the outward budding of cell membrane is microvesicles which their sizes are 100–1000 nm [3]. Microvesicles are also called shedding vesicles, microparticles, shedding bodies, ectosomes and oncosomes. A number of functions are attributed to microvesicles such as intercellular signaling and changing the extracellular environment. They also facilitate cell invasion through cell-independent matrix proteolysis [32]. Microvesicles, same as exosomes, carry mRNA, short interfering RNA (siRNA) and ectopically expressed reporter proteins, but it has been shown that plasmid DNAs, which have reporter functions, could only be transferred to target cells by microvesicles [32, 33]. Researches demonstrated that microvesicles have also crucial roles in stem cell expansion and renewal [34], tumor progression [35, 36], coagulation [37] and inflammation [38].

Apoptotic bodies are formed via the membrane blebbing of apoptotic cells. Their usual size is more than 1000 nm [39]. As far as we know to date, no therapeutic effect of apoptotic bodies has been seen in eye diseases [3]. However, exosomes have noteworthy therapeutic effects against many diseases including neurologic ones [40–42]. MSC-derived exosomes’ (MSC-Exo) neuroprotective effect was also discovered in retinal cell injuries such as retinal cell degeneration, refractory macular holes, retinal detachment and optic nerve injury. MSC-Exos could reduce cell apoptosis and restrict the area of the injury in these diseases [27].

The main reason that why the EVs have become a research interest is their inner load which contain mRNAs, miRNAs, lipids and proteins. EVs’ cell signaling task is done by these components [3]. Many studies have shown that mRNAs and miRNAs play important roles in this task. While mRNAs can induce translation of new proteins in target cells, miRNAs can regulate the expression of genes [43, 44]. EVs’ multiple therapeutic effects are done by entering mRNAs, miRNAs and proteins into target cells [3]. MSC-EVs express adhesion molecules such as CD29, CD73 and CD44 which allow them to adhere to the damaged and inflamed sites of tissues [21]. Considering the source of EVs, their inner components vary. The two other factors which also influence the inner cargo and subsequently the therapeutic effects of exosomes are the source cell passages and its phase of differentiation [3]. It has been shown that the neuroprotective efficacy of MSC-Exos reduces with raising cell passages [45]. It has also been indicated that exosomes’ cargos vary at different stages of their source cell differentiation. For instance, exosomal miRNAs were differentially expressed in distinct stages of BMSCs osteogenic differentiation [46]. The composition of EVs’ cargos is not just a sample of the cytoplasm of their cell of origin. Studies demonstrated that some proteins, mRNAs, miRNAs and transfer RNAs are more abundant in EVs than the cytoplasm of their original cells [47–49].

Ocular therapies which are based on EVs have many advantages over cell-based therapies. Retina MSC-based therapy has incurred safety concerns. For example, a report showed that three patients with AMD who underwent intravitreal injection of adipose-derived MSCs, became blind because of the hemorrhage and retinal detachment [50]. One explanation for these pathologies is the adherence of transplanted MSCs to the inner limiting membrane of retina that would make an epiretinal membrane [51–53]. Another explanation would be the possible result of undesired differentiation of transplanted MSCs [20]. Other complications of cell therapy are the lack of information of the rate of cell death and cell division after administration [54]. Moreover, an important downside of cell therapy in retina is that the transplanted cells would not become integrated into the retina efficiently [13, 55]. The occasionally cell integration will be done through the digestion of inner limiting membrane and retinal glial activity modulation that might damage the retina themselves [22]. Since many studies have shown that keeping the therapeutic benefits of cell therapy, the EV therapy would avoid most of the above complications and also some EVs can cross the inner limiting membrane freely, it would be a better choice than cell therapy [12, 15].

## miRNAs

**Fig. 1:**
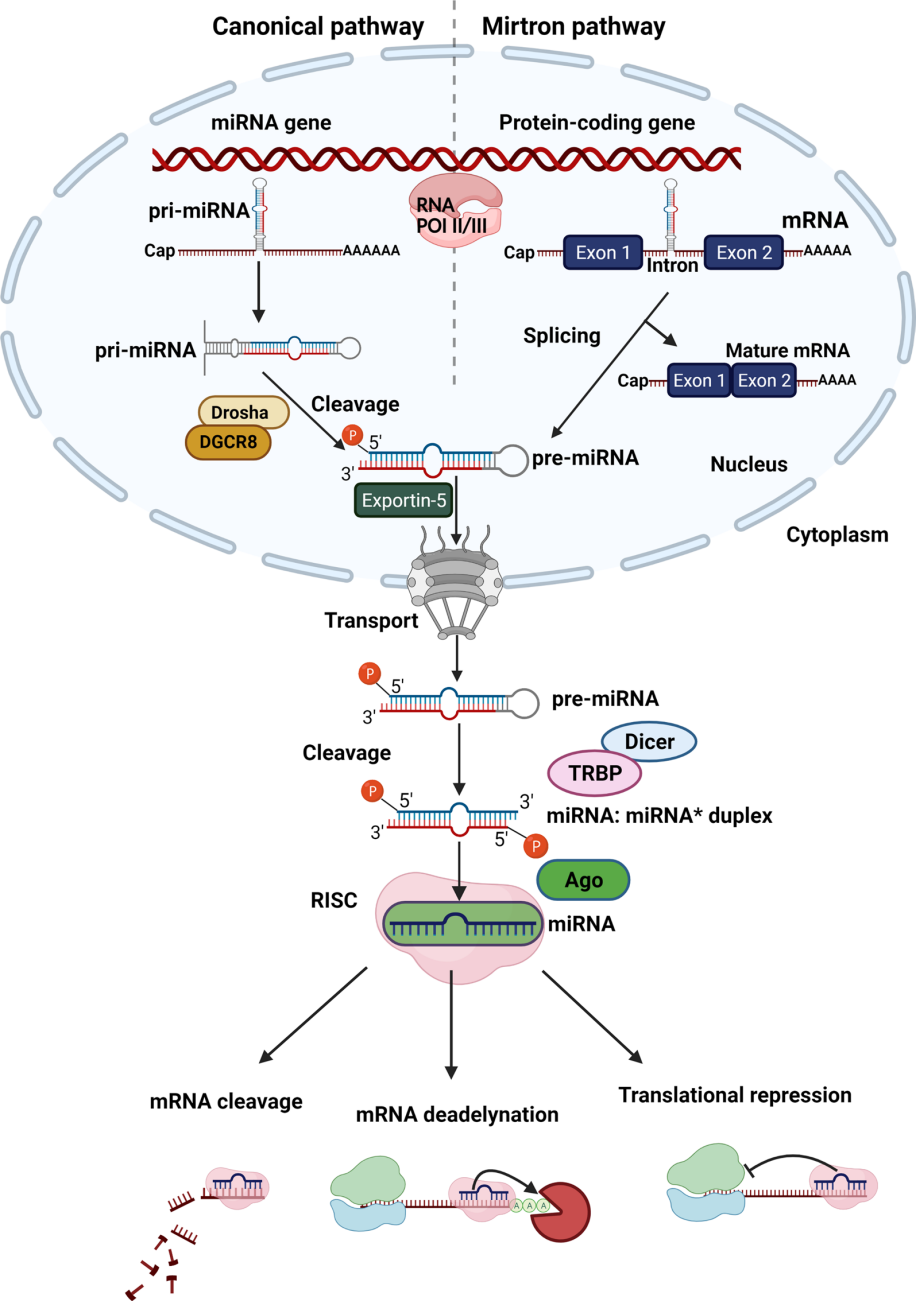
MiRNA synthesis pathway. Biogenesis of miRNA begins with transcription of a miRNA gene (Canonical pathway) or the intron region of a protein-coding gene (Mirtron pathway) mainly by RNA polymerase II, and sometimes by RNA polymerase III in the nucleus. Canonical pathway: The sequences from miRNA genes transcription self-anneal and make hairpin-like structures called primary miRNAs (pri-miRNAs). Pri-miRNAs are being cut by DGCR8/Drosha complex and become pre-miRNAs. Mirtron pathway: Pre-miRNAs which are the result of intron regions of protein-coding genes are not dependent on Drosha complex. They are divided by spliceosome from the primary transcript of mRNAs. Then, they will self-anneal and become pre-miRNAs directly. All Pre-miRNAs from both pathways leave the nucleus and enter the cytoplasm by Exportin-5. There, the pre-miRNAs are cleaved by the Dicer/TRBP complex, yielding an about 22 nucleotides long miRNA: miRNA* duplex molecule. Then, this molecule will be loaded into the Argonaute (Ago) part of RNA-induced silencing complex (RISC). After discarding one of the strands, the other one will remain in the RISC and binds to 3’ untranslated regions of target mRNAs. miRNAs binding to target mRNAs lead to their translational repression, deadenylation and cleavage

miRNAs are a subdivision of evolutionary conserved long non-coding RNAs with approximately 22 nucleotides and a post-transcriptive repressive influence on gene expression [56–58]. First step in the biogenesis of miRNAs is the production of partially complementary primary RNA transcripts (pri-miRNA) mostly by RNA polymerase II and sometimes by RNA polymerase III. miRNAs will derive from these structures. Pri-miRNAs become hairpin structures by self-annealing. Then, the miRNA processing complex, which is made of Drosha ribonuclease and the DiGeorge Critical Region 8 (Dgcr8) proteins, will make a cut in the hairpin structure at the end of 11 base pairs (bp) from the foundation of the hairpin stem [59]. A seventy nucleotide sequence called precursor miRNA (pre-miRNA) will be released as a result [56]. The pre-miRNA is transferred to the cytoplasm by Exportin-5. Then, the Dicer endoribonuclease will attach to the pre-miRNA and cleave it to release a ~ 22 nucleotide long double strand RNA named miRNA* duplex. Since the pre-miRNA itself has a 5′ phosphate at one end and a 3′ two-nucleotides’ overhang at the other end, the dicer cleavage makes one phosphate at the 5′ end of each new strand, and a two-nucleotides’ overhang at the 3′ end of each new strand. Afterward, the miRNA* duplex will be incorporated into the Argonaute protein (Ago) which is a part of the RNA-induced silencing complex (RISC) and one strand will be removed. The remaining strand that is connected to RISC will attach partially to target mRNAs and repress their translation or induce degradation (Fig. 1). One miRNA can bind to myriads of target mRNAs [56, 60, 61].

miRNA nomenclature is based on an annotation system which was introduced by Ambros et al. [62]. In brief, miRNA genes are numbered by the sequence of their discovery. Identical or nearly identical miRNAs from different species get the same number. A miRNA number is always accompanied by a prefix: mir or miR. The pre-miRNA is shown by “mir” prefix and the mature miRNA is preceded by “miR.” They are followed by a dash and then the number comes (e.g., mir-25 and miR-25). Identical mature miRNAs with one or two different nucleotides in their sequences are distinct by a lower case letter (e.g., miR-36a and miR-36b). A dash and a number suffix will be added to the names of pre-miRNAs that make identical mature miRNAs despite locating on different loci of the genome (e.g., mir-42a-1 and mir-42a-2 produce an identical mature miRNA, miR-42a). In the miRNA formation process, a miRNA duplex will be cleaved to two different mature miRNA strands: the one that comes from the 5′ arm is shown by 5p (e.g., miR-146b-5p) and the one from the 3′ arm by 3p (e.g., miR-146b-3p). Having said that, if the relative level of cell abundance of same miRNAs’ two strands is known, the arm with the lower expression will get an asterisk following the number (for instance miR-9 is more abundant than miR-9*). miRNA names can also indicate the species of origin by a three-letter prefix: for example, “hsa” stands for *Homo sapiens* in hsa-miR-132 and “rno” for *Rattus norvegicus* in rno-miR-125 [62, 63].

**Fig. 2:**
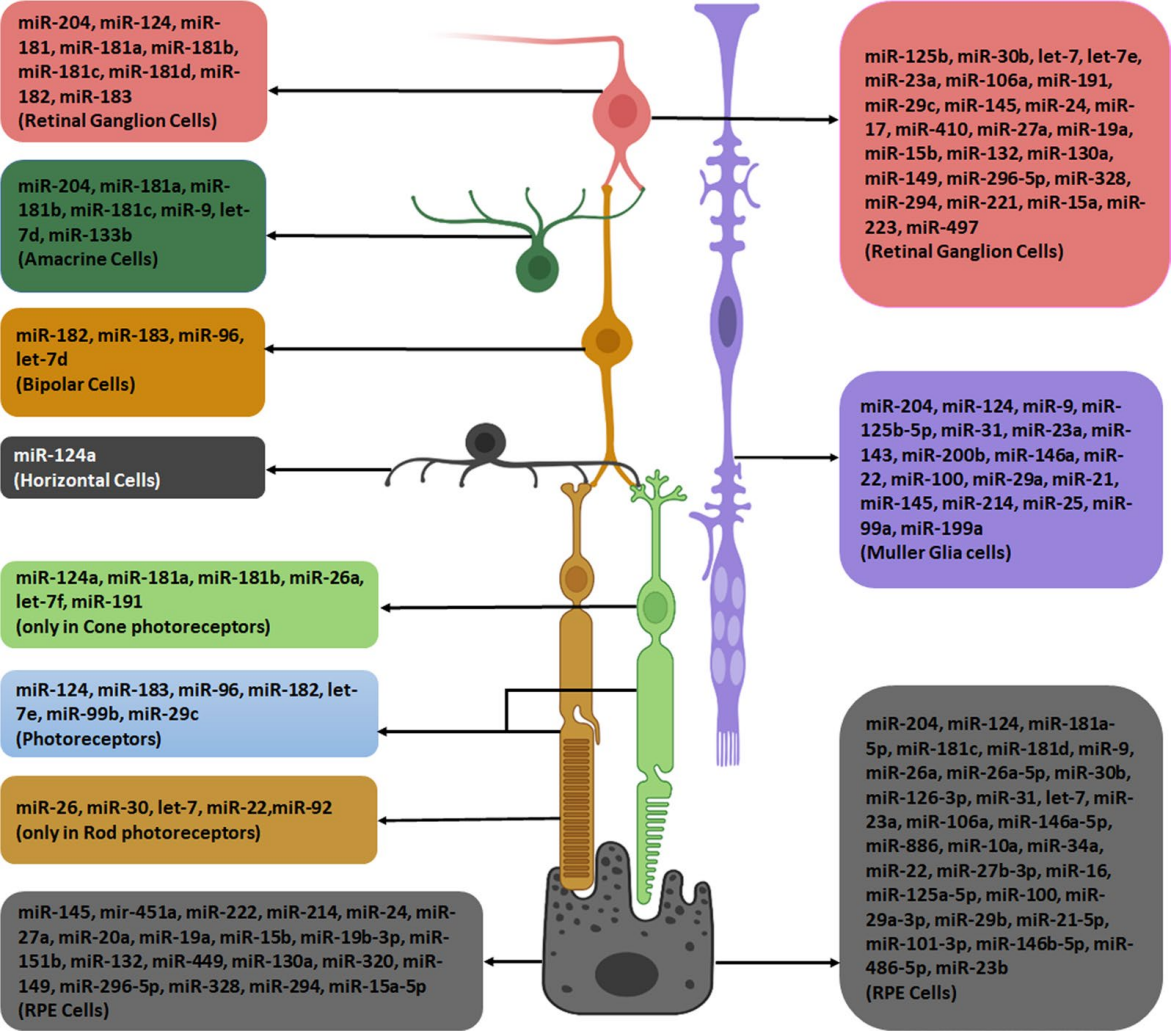
MiRNAs enriched in retinal cells which are also present in MSC-EVs

Defects in miRNAs synthesis can make serious problems in the development process and is related to pathologies including inherited genetic disorders, diabetes, cancers, heart failure and neurodegenerative diseases. miRNAs maintain the healthy condition of gene networks and modulate the ups and downs of gene expression in developed tissues [56]. As well as other tissues, miRNAs play important roles in retina and some of them are more enriched in retinal cells (Fig. 2) [64]. Many studies showed their role in the function and survival of different retinal cells such as photoreceptors or Müller glias [65, 66]. Here, we discuss retinal cell miRNAs (Table 1) similarities with MSCs-EVs’ miRNAs (Table 2) and their possible therapeutic effects on retinal diseases.

**Table 1:** miRNAs of retina

Retina miRNAs	References	Retina miRNAs	References
miR-204	[60, 64–66, 90–107]	miR-142b	[66, 108, 109]
miR-124a	[64, 90, 93, 95, 98, 99, 101, 104, 105, 110, 111]	miR-7a	[66, 107–109, 112]
miR-9	[65, 66, 90, 92, 94, 95, 99, 101, 103, 105, 107, 108, 111, 113–117]	miR-27c	[66, 108, 109]
miR-9*	[66, 90, 99, 107, 108]	miR-25	[97, 107, 108]
miR-29	[90, 95]	miR-133	[95]
miR-181a	[60, 90, 94, 95, 98–101, 105–107, 118–120]	miR-1	[95]
miR-182	[60, 64, 65, 90, 93–95, 97–101, 103–107, 111, 120–122]	miR-185	[95, 97]
miR-183	[60, 64, 65, 90, 93–95, 97–101, 104, 106, 107, 111, 120–122]	miR-219	[95]
miR-183*	[106, 107]	miR-124a-1	[65]
miR-125b	[90, 92, 98, 99, 107, 113, 123, 124]	miR-132	[65, 99, 101, 107]
miR-26a	[90, 98, 107, 120, 123]	miR-23a	[65, 66, 101, 107, 123, 125]
miR-181	[90]	miR-449a	[126]
miR-96	[60, 64, 65, 90, 93–95, 97, 99–101, 104, 106, 107, 121, 122]	miR-449b-5p	[126]
let-7	[65, 66, 90, 93, 94, 98, 113–115, 117]	miR-9–1	[97]
let-7i	[90, 107, 125]	miR-181b-1	[97]
miR-106b	[90, 97, 101, 107, 127]	miR-181a-1	[97]
miR-30b	[90, 92, 101]	miR-181a-1*	[107]
miR-139	[90, 125]	miR-29c	[64, 97, 99, 101, 105, 107]
miR-126	[90, 128]	miR-194–1	[97]
miR-107	[90]	miR-194–2	[97]
miR-103	[90, 107]	miR-7–2	[97]
miR-422a	[90]	miR-9–3	[97]
miR-422b	[90]	miR-181-c	[97]
miR-335	[90, 95, 97]	miR-181-d	[97]
miR-31	[66, 90, 97, 101, 108, 109]	miR-7–3	[97]
miR-106	[66, 90]	miR-216b	[97]
miR-129-3p	[90, 100, 101, 107, 129]	miR-217	[97, 99]
miR-691	[90, 107]	miR-9–2	[97]
miR-26b	[90, 107, 123]	miR-219–1	[97]
miR-35	[90]	miR-30c	[98, 101]
miR-886-5p	[91]	miR-213	[99]
miR-184	[65, 91, 94, 97, 99, 101, 126, 130]	miR-454a	[99]
miR-146a	[66, 91, 108, 109, 131]	let-7d	[95, 99, 101, 103, 107, 123]
miR-10a	[91]	miR-205	[99]
miR-203	[66, 91, 132]	let-7b	[64, 99, 100, 107, 123]
miR-194	[91, 95]	miR-130a-3p	[133]
miR-200b	[128, 134]	miR-20a-5p	[124, 133]
miR-200b*	[107]	miR-93-5p	[133]
miR-34a	[65, 107, 135]	miR-9-3p	[133]
miR-182-5p	[136]	miR-709	[107, 133]
miR-183-5p	[136]	let-7a	[66, 107, 123, 124]
miR-26a-5p	[124, 136]	miR-16	[107, 123, 137]
miR-181a-5p	[124, 136]	miR-320	[107, 123]
miR-204-5p	[124, 136]	let-7e	[101, 107, 123]
miR-22-3p	[136]	miR-7	[65, 138]
let-7a-5p	[124, 136]	miR-200c	[101]
miR-191-5p	[136]	miR-221	[101]
miR-124-3p	[136]	miR-33	[101, 107]
miR-9-5p	[133, 136]	miR-342-3p	[101]
miR-127-3p	[136]	miR-365	[101]
miR-192-5p	[136]	miR-467a	[101]
let-7f-5p	[124, 136]	miR-470	[101]
miR-27b-3p	[124, 136]	miR-542-3p	[101]
miR-96-5p	[136]	miR-652	[101]
miR-26b-5p	[136]	miR-695	[101]
miR-30b-5p	[124, 136]	miR-774	[101]
miR-92a-3p	[133, 136]	miR-375	[101]
miR-99b-5p	[136]	miR-465c-5p	[101]
miR-125b-5p	[66, 124, 136]	miR-30a	[101, 107]
miR-151a-5p	[136]	miR-15a	[101, 107]
miR-211-5p	[124, 136]	miR-223	[101]
miR-126-5p	[136]	miR-290-5p	[101, 107]
miR-143-3p	[136]	miR-29b	[101, 107, 139, 140]
miR-16-5p	[124, 136]	miR-379	[101]
let-7 g-5p	[124, 136]	miR-380-3p	[101]
miR-148a-3p	[136]	miR-384-5p	[101]
miR-181b-5p	[136]	miR-409-5p	[101]
miR-125a-5p	[107, 124, 136]	miR-433	[101]
miR-92b-3p	[136]	miR-497	[101]
miR-181a-2-3p	[136]	miR-541	[101]
miR-181c-5p	[136]	miR-551b	[101, 107]
miR-30d-5p	[124, 136]	miR-676	[101]
miR-100-5p	[136]	miR-713	[101, 107]
let-7c-5p	[136]	miR-742	[101]
miR-103a-3p	[124, 136]	miR-875-3p	[101]
miR-29b-3p	[136]	miR-378	[101]
miR-151a-3p	[136]	miR-465b-5p	[101]
miR-186-5p	[136]	miR-28	[60, 141]
miR-21-5p	[124, 136]	miR-145	[66, 101, 111, 142]
miR-30a-5p	[99, 124, 136]	miR-149	[101]
miR-146a-5p	[136]	miR-188-5p	[101]
miR-101-3p	[124, 136]	miR-339-5p	[101]
miR-126-3p	[101, 136]	miR-130a	[101, 107]
miR-146b-5p	[136]	miR-883b-5p	[101]
miR-266-5p	[136]	miR-490	[101]
miR-486-5p	[136]	miR-381	[101]
miR-99a-5p	[136]	miR-680	[101]
miR-23b-3p	[124, 136]	miR-882	[101]
miR-30e-5p	[136]	miR-500	[101]
let-7b-5p	[136]	miR-495	[101]
miR-10a-5p	[136]	miR-335-5p	[101]
miR-27a-3p	[124, 136]	miR-296-5p	[101]
miR-29a-3p	[136]	miR-328	[101]
miR-181a-3p	[136]	miR-294	[101]
miR-142-5p	[136]	miR-467e	[101]
miR-145-5p	[136]	miR-329	[101]
miR-451a	[136]	miR-466d-3p	[101]
miR-23a-3p	[124, 136]	miR-34c	[101]
miR-124	[60, 66, 92–94, 107, 108, 114, 133, 143]	miR-484	[101]
miR-125a	[92, 125]	miR-191	[101, 107, 120]
miR-762	[144]	miR-382	[101]
miR-24a	[93, 104, 114, 145]	miR-468	[101]
miR-133b	[93]	miR-681	[101]
miR-218	[93, 101]	miR-455	[101]
miR-196a	[93]	miR-99a	[66]
miR-129	[93, 104, 117, 144]	miR-135a	[66, 107]
miR-222	[93, 104, 117, 125, 144]	miR-21	[66, 128]
miR-214	[93, 104, 111, 117, 125, 128, 144]	miR-29a	[66, 107, 111, 146]
miR-155	[93, 99, 104, 117, 144, 147]	miR-143	[66, 107, 111]
miR-210	[94, 97, 106, 107]	miR-199a-3p	[66]
miR-140	[94, 106, 107]	miR-199a-5p	[66]
miR-211	[60, 64, 65, 94, 96, 100, 102]	miR-199b	[66]
miR-181b	[60, 94, 95, 99, 101, 106, 107, 118, 120]	miR-199b*	[66]
let-7f	[94, 107, 120]	miR-17-5p	[128]
miR-22	[66, 94, 107, 125]	let-7e-5p	[124]
miR-26	[94]	miR-19b-3p	[124]
miR-30	[94]	miR-19a-3p	[124]
miR-92	[94, 95]	miR-106b-5p	[124]
miR-125	[65, 66, 94, 114, 115, 117]	miR-15a-5p	[124]
miR-34	[132]	miR-455-3p	[124]
miR-350	[101, 132]	miR-34a-5p	[124]
miR-410	[101, 132]	miR-24-3p	[124]
miR-216	[99, 132]	miR-30c-5p	[124]
miR-212	[107, 132]	miR-301b	[111]
miR-181c	[95, 101, 111, 129]	miR-199	[111]
miR-181c*	[129]	miR-27b	[107]
miR-129-5p	[129]	miR-338-3p	[107]
miR-99b	[101, 107, 129]	miR-138	[107]
miR-23b	[98, 107, 123, 129]	miR-127	[107]
miR-24	[101, 107, 123, 129]	miR-151-5p	[107]
miR-30d	[101, 129]	miR-193	[107]
miR-503	[101, 129]	miR-136	[107]
miR-27a	[101, 107, 129]	miR-195	[107]
miR-135	[148]	miR-148a	[106, 107]
miR-18a	[107, 127, 128, 149]	miR-452	[107]
miR-130b	[127]	miR-542	[107]
miR-20a	[107, 127, 128]	miR-292-5p	[107]
miR-34b-5p	[127]	miR-744	[107]
miR-216a	[66, 97, 127]	miR-689	[107]
miR-20b	[107, 127]	miR-423-5p	[107]
miR-17	[66, 101, 107, 127, 150]	miR-677	[107]
miR-18b	[127]	miR-301a	[107]
miR-106a	[101, 107, 127]	miR-130b	[107]
miR-19a	[99, 107, 127]	miR-374	[107]
miR-93	[107, 127]	miR-32	[107]
miR-15b	[101, 107, 123, 127, 137]	miR-146b	[107]
let-7a-2	[125]	miR-153	[107]
let-7c	[107, 125]	miR-19b	[107]
let-7f-2	[125]	miR-207	[107]
miR-100	[66, 125, 129]	miR-489	[107]
miR-125b-1	[125]	miR-700	[107]
miR-125b-2	[125]	miR-92b	[99, 107]
miR-151b	[125]	miR-101a	[107]
miR-152	[101, 125]	miR-690	[107]
miR-181d	[101, 125]	miR-720	[107]
miR-26a-1	[125]	miR-7b	[107]
miR-26a-2	[125]	miR-361	[97]
miR-3120	[125]	miR-181a-2	[97]
miR-4521	[125]	miR-181b-2	[97]
miR-98	[95, 107, 125]	miR-219–2	[97]
miR-206	[90]	miR-7–1	[97]
miR-150	[151]	–	–

**Table 2:** miRNAs of MSC-EVs

MSCs’ EVs miRNAs	References	MSCs’ EVs miRNAs	References
miR-146a	[21, 61, 152–161]	miR-494	[156–158, 162]
miR-155	[152, 158]	miR-140-5p	[162]
miR-21	[21, 40, 152–154, 156, 158–160, 163–165]	miR-196a	[61]
miR-27b	[152, 158]	miR-27a	[61]
let-7	[152]	miR-206	[61, 166]
miR-126	[61, 152, 156, 160, 167, 168]	miR-199a	[61, 156, 165]
miR-886	[152]	miR-302a	[61, 159]
miR-22	[21, 40, 42, 61, 70, 154, 156, 164, 169, 170]	miR-133	[61, 70]
miR-133b	[40, 42, 61, 156, 157, 163, 164, 166, 169, 171]	miR-155-5p	[61]
miR-19a	[21, 40, 70, 156, 169]	miR-16-5p	[61, 83, 172, 173]
miR-100	[153, 154, 156, 159, 165, 174]	miR-223-3p	[61]
miR-143	[42, 153, 154, 158, 163]	miR-15a	[61]
miR-181	[70, 153, 160, 161]	miR-15b	[61]
miR-221	[40, 153, 154, 156, 157, 165, 174]	miR-125a-3p	[61]
miR-145-5p	[70, 83, 153, 172, 175]	miR-142-3p	[61, 83, 173, 174]
miR-16	[61, 156, 157, 165, 170, 174]	miR-223	[61, 70, 156, 158, 174]
miR-17	[21, 156]	miR-630	[155]
miR-130a	[156, 160, 167]	miR-204	[166]
miR-132	[154, 156, 160, 167]	miR-328	[166]
let-7b	[21, 154, 156, 158, 160, 161, 167, 168]	miR-210	[40, 156, 159]
let-7c	[21, 70, 154, 156, 160, 167]	miR-23a-3p	[70, 83, 88, 173, 175]
miR-486-5p	[3, 70, 82, 88]	miR-1260b	[70, 165, 175]
miR-10a-5p	[70, 82]	miR-1246	[3, 70, 83]
miR-10b-5p	[70, 82, 88]	miR-451a	[70, 83]
miR-191-5p	[70, 82]	miR-4454	[70, 83]
miR-222-3p	[70, 82, 83, 173]	miR-21a-5p	[70]
miR-143-3p	[70, 82, 83, 88]	miR-486b-5p	[70]
miR-22-3p	[70, 82, 83, 88]	miR-486a-3p	[70]
miR-21-5p	[3, 21, 61, 70, 82, 83, 88, 156, 172, 173, 175]	miR-486a-5p	[70]
let-7a-5p	[3, 70, 82, 83, 172, 173, 175]	miR-486b-3p	[70]
miR-127-3p	[21, 82, 83]	miR-125a	[156, 174]
miR-99b-5p	[82]	miR-1792	[156]
miR-100-5p	[70, 82, 83, 88, 172, 173, 175]	miR-1587	[156]
miR-92a-3p	[3, 70, 82, 172]	miR-124a	[156]
miR-26a-5p	[82, 156]	miR-101-3p	[156]
miR-146a-5p	[82]	miR-23b-5p	[156]
miR-4485	[82]	miR-339-3p	[156]
miR-146b-5p	[82]	miR-425-5p	[156]
miR-151a-3p	[82]	miR-34a	[156]
let-7f-5p	[70, 82, 88, 175]	miR-210-3p	[156]
miR-92b-3p	[82]	miR-294	[156]
miR-423-5p	[3, 82]	miR-133b-3p	[156]
miR-27b-3p	[82, 83]	miR-200b	[156]
let-7i-5p	[82]	miR-99a	[174]
miR-28-3p	[82]	miR-627	[174]
miR-125b-5p	[21, 61, 70, 82, 83, 88, 159, 172, 173, 175]	miR-142-5p	[174]
miR-19b	[174]	miR-383	[174]
miR-124	[154, 163]	miR-501	[174]
miR-233	[21]	miR-601	[174]
miR-181-5p	[21]	miR-17-3p	[174]
miR-145	[21, 154, 156, 159, 161, 164, 165]	miR-497	[176]
miR-223-5p	[21]	miR-486	[174]
miR-30	[21, 61, 70]	miR-451	[174]
miR-92a	[154]	miR-564	[174]
miR-146	[21]	miR-30a	[158]
miR-30b	[156, 168]	miR-410	[159, 161]
miR-181c	[158, 159, 161, 168]	miR-181b	[161]
miR-126-3p	[61, 168]	miR-181d	[161]
miR-4484	[168]	miR-1252	[161]
miR-619-5p	[168]	miR-4434	[161]
miR-6879-5p	[168]	miR-4669	[161]
miR-291a-3p	[168]	miR-199b-3p	[83]
miR-23b	[42, 70, 154, 156, 158, 164]	miR-7975	[83]
miR-122	[40, 70, 154]	let-7b-5p	[83]
miR-1224-5p	[154]	miR-29a-3p	[83]
miR-1228	[154]	miR-144-3p	[83]
miR-1234	[154]	miR-29b-3p	[83]
miR-1237	[154]	miR-630	[83]
miR-1238	[154]	miR-221-3p	[3, 83, 173]
miR-150*	[154]	let-7i-5p	[83]
let-7b*	[154]	miR-424-5p	[83]
let-7d*	[154]	miR-191-5p	[83]
miR-198	[154]	miR-25-3p	[83, 172]
miR-296-5p	[154]	miR-130a-3p	[83]
miR-572	[154]	miR-376a-3p	[83]
miR-765	[154]	miR-4286	[83]
miR-933	[154]	miR-15a-5p	[83]
miR-149	[154]	miR-24-3p	[83, 172, 173]
miR-149*	[154]	miR-34a-5p	[83]
miR-191	[154, 165]	miR-122-5p	[3, 83]
miR-191*	[154]	miR-181a-5p	[83]
miR-425*	[154]	miR-199a-5p	[83]
miR-574-5p	[154]	miR-495-3p	[83]
miR-575	[154]	miR-196a-5p	[83]
miR-638	[154]	miR-320e	[83]
miR-663	[154]	miR-148a-3p	[83]
miR-671-5p	[154]	miR-93-5p	[83]
miR-923	[154]	miR-377-3p	[83]
miR-940	[154]	miR-382-5p	[83]
let-7a	[154, 156, 158, 165]	miR-15b-5p	[83]
let-7d	[154]	miR-376c-3p	[83]
let-7e	[154, 156]	miR-374a-5p	[83]
let-7f	[154, 156, 165]	let-7e-5p	[83]
let-7i	[154]	miR-379-5p	[83]
miR-103	[154]	let-7c-5p	[83]
miR-107	[154]	miR-1260a	[165, 175]
miR-125a-5p	[83, 154]	miR-320a	[3]
miR-125b	[40, 154, 156, 159, 164, 165, 174]	miR-195	[165]
miR-151-5p	[154, 156]	miR-106a-5p	[172]
miR-181a	[154, 158, 161]	miR-19b-3p	[172]
miR-199a-3p	[70, 83, 154, 161, 175]	miR-320	[154]
miR-214	[154]	miR-361-5p	[154]
miR-222	[154, 165, 174]	miR-574-3p	[154]
miR-23a	[154, 156, 159, 165]	miR-26a	[154]
miR-24	[154, 174]	miR-17–92 cluster: (miR-17, miR-18a, miR-19a, miR-19b, miR-20a and miR-92a)	[12, 21, 40, 70, 177]
miR-31	[154, 174]	miR-23b-3p	[178]

## miRNAs of EVs

Literatures have shown different procedures of loading miRNAs into EVs. Some studies demonstrated that when MVBs bind to plasma membrane and EVs are made, RISC complex is associated with them [67, 68]. Other studies which concluded that RISC or Argonaute2 (Ago2) is not present in EVs indicated that packing miRNAs takes place by a type of ubiquitous proteins called heterogeneous nuclear ribonucleoproteins (hnRNP) [69]. Some motifs of miRNAs either alone or associated with proteins such as Ago2, Alix and MEX3C can be detected by and attached to hnRNP [70]. For instance, the loading of GGAG motif of miRNAs into EVs is controlled by the attached nuclear hnRNPA2B1 (ribonucleoprotein A2B1) [71].

Other proteins such as synaptotagmin-binding cytoplasmic RNA-interacting protein (SYNCRIP) detect miRNAs’ motifs which bind to the GGCU motif [72]. As a study showed that the mutation in Alix protein diminishes miRNAs levels in EVs, it can be concluded that this protein is also important in packing miRNAs into EVs [61, 73].

EVs inner cargos enter the target cells by two methods: endocytosis and fusion [70]. EVs are mainly taken up by endocytosis, according to previous studies [74–77]. Clathrin-dependent endocytosis and clathrin-independent pathways that are mediated by caveolin, phagocytosis, macropinocytosis and lipid raft-mediated uptake are different types of this mechanism [74]. Considering the cell types and components of EVs, a group of them may be absorbed by more than one mechanism[78]. The direct fusion of EVs’ membrane with cell membrane is the second mechanism of EVs entering into the target cells [79]. It was reported that spontaneous transfer of EVs took place between dendritic cells by fusion and release of the inner cargo into the cytoplasmic matrix [75].

Many literatures demonstrated that EVs miRNAs may affect target cells. Valadi et al. made the first report on evident transfer and function of mRNAs and miRNAs of EVs. They found new mouse proteins in the target cells after conveying the cargo of mouse EVs to human mast cells [44].

In addition, Song et al. indicated the transfer of functional miRNAs of MSC-EVs. After treating MSCs with IL-1β, the expression of miR-146a increased. Then, miR-146a was packaged into EVs selectively. As a result of co-culturing the MSC-EVs with macrophages, the level of miR-146a in macrophages had been raised which led to M2 polarization [80].

Many studies have shown the differences of miRNAs between EVs and their parental MSCs. A research showed that the expression of mir-15 and mir-21 was significantly higher in MSCs than their EVs [81]. Baglio et al. manifested that the miR-34a-5p, miR-34c-5p, miR-15a-5p and miR-136-3p are more represented in MSCs than their EVs and miR-4485, miR-150-5p, miR-6087 and miR-486-5p are enriched in MSC-EVs compared to MSCs [82].

There are differences among MSC-EVs’ miRNAs from various sources. Baglio et al. compared the miRNA contents of EVs derived from bone marrow and adipose MSCs. Most abundant miRNAs of bone marrow-derived MSC-EVs were miR-143-3p, miR-10b-5p, miR-486-5p, miR-22-3p and miR-21-5p, whereas, miR-486-5p, miR-10a-5p, miR-10b-5p, miR-191-5p and miR-222-3p were the most frequent miRNAs of adipose-derived MSC-EVs [82]. 171 miRNAs of hBMSC-EVs were disclosed in another research. While 148 miRNAs constitute 0.03 to 0.7% of the total reads, the 23 most abundant miRNAs made up 79.1% of them [83]. Luther et al. showed that the highest expressed EVs miRNA of mouse bone marrow-derived MSCs is miR-21a-5p which is responsible for MSCs cardioprotection [84]. The variety of miRNA profile among MSC-EVs may suggest that the expression of miRNAs is due to multiple factors and the effects of MSC-EVs may be the result of each miRNA synergistical activity with other elements [70]. MSC-EVs’ miRNAs are provided in Table 2.

## MSCs’ miRNAs potential therapeutic effects

Over the last years, the effects of many miRNAs on retinal cells development and function have been revealed and the expression of miRNAs in normal and pathological conditions have been investigated. MSC-EVs contain some miRNAs which their roles in retinal cells’ function and development have been proved, so studying them as therapeutic agents for retinal neurodegenerative diseases has not been overlooked.

**Fig. 3:**
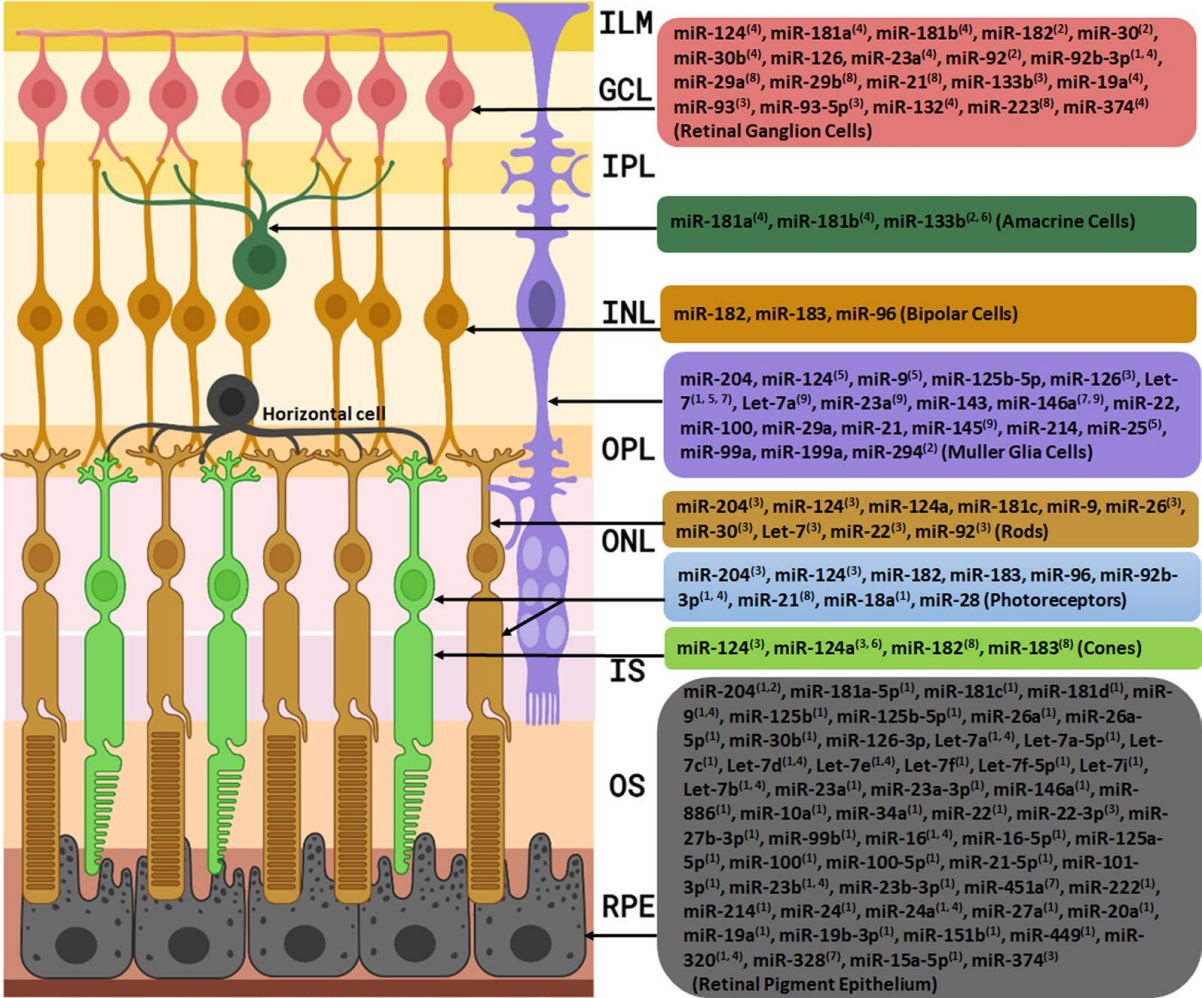
MSC-EVs’ miRNAs with studied effects on retinal cells. ILM, inner limiting membrane; GCL, ganglion cell layer; IPL, inner plexiform layer; INL, inner nuclear layer; OPL, outer plexiform layer; ONL, outer nuclear layer; IS, inner segment of photoreceptors; OS, outer segment of photoreceptors; RPE, retinal pigment epithelium. General effects of miRNAs on retinal cells: ^1^differentiaition, ^2^function, ^3^survival & apoptosis reduction, ^4^development & growth, ^5^reprogramming, ^6^maturation, ^7^proliferation, ^8^protection & maintenance, ^9^dedifferentiation

Therapeutic effects of a number of MSC-EVs’ miRNAs on retinal degenerative diseases have been assessed (Fig. 3). For example, Mead and Tomarev showed that by knocking down the Ago2 which plays a critical role in regulating the biological function of miRNA and the consequent reduction of miRNA abundance in exosomes, the BMSC-derived exosomes (BMSC-Exos) had lost their effects in advancing RGC neuroprotection, axon viability/regeneration and RGC functional maintenance [12]. They concluded that while knocking down Ago2 does not have an influence on exosomes’ protein content, the above results demonstrated the dependency of RGC treatment on miRNA in comparison to the protein. BMSC-derived exosomes contain miR-17-92 which can downregulate phosphatase and tensin homolog (PTEN) expression [85]. As PTEN expression is a major suppressor of RGC axonal growth and survival [86, 87], RGC neuroprotection was done probably by miR-17-92 [12]. miR-21 and miR-146a which were identified in exosomes of umbilical cord MSCs and BMSCs, respectively, may be another candidates of RGC protection and survival [12, 88]. In another study, Zhang et al. showed that MSC exosomes containing miR-126 ameliorate the inflammation and promote vascular repair in diabetic retinopathy (DR). They indicated that miR-126 reduces the inflammation in diabetic rats by inhibiting HMGB1 signaling pathway [89].

Having knowledge of the similarities between miRNAs that have an effect on retinal cells development and function and the miRNA content of MSC-EVs, we can design research and therapies more effectively and specifically for retinal degenerative diseases. Functions of miRNAs in retina can be divided into different categories. Many of them take part in differentiation process (e.g., miR-204, miR-124, miR-30b, miR-133b, …), a remarkable number in development (e.g., miR-181, miR-126, miR-155, miR-17, …), and a group of them in cell proliferation (e.g., miR-103, miR-124, miR-34a, miR-15b, …). Some of them will decrease cell apoptosis and contribute to cell survival and maintenance (e.g., miR-30, miR-124, miR-22, miR-29a, …) while a few participate in neurons’ connectivity and plasticity (miR-124, miR-133b, miR-132). Moreover, therapeutic effects of a number of miRNAs have been discovered in some of retinal diseases. miR-200b, miR-148a-3p and miR-15a act against DR while miR-361, miR-497 and miR-140 are retinoblastoma tumor suppressors. It had also been reported that miR-222 can prevent the progression of retinal degeneration and miR-124 has therapeutic effects on it. A few miRNAs have various proven functions in retina: for instance, miR-204 plays roles in differentiation, development and decreasing apoptosis whereas miR-124 has effects on differentiation, proliferation, survival of photoreceptors, plasticity and connectivity of neurons and a studied positive effect on retinal degeneration. The data are presented in detail in Table 3.

**Table 3:** MSC-EVs and retina common miRNAs; their expression, sequences and effects

miRNA	Retina expression patterns	Sample	miRNA sequence, miRBase accession number	Effect	Retina ref	EV ref
miR-204	RPE, amacrine cells, INL, ONL, GCL (adult), Müller glia, mature retina	Human, mouse, medaka fish, zebrafish, rat	> hsa-miR-204-5p MIMAT0000265: UUCCCUUUGUCAUCCUAUGCCU > mmu-miR-204-5p MIMAT0000237: UUCCCUUUGUCAUCCUAUGCCU > ola-miR-204 MIMAT0022589: UUCCCUUUGUCAUCCUAUGC > dre-miR-204-5p MIMAT0001279 UUCCCUUUGUCAUCCUAUGCCU > rno-miR-204-5p MIMAT0000877 UUCCCUUUGUCAUCCUAUGCCU > hsa-miR-204-3p MIMAT0022693: GCUGGGAAGGCAAAGGGACGU > mmu-miR-204-3p MIMAT0017002: GCUGGGAAGGCAAAGGGACGU > dre-miR-204-3p MIMAT0031924 GGCUGGGAAGUCAAAGGGACGC > rno-miR-204-3p MIMAT0004739 GCUGGGAAGGCAAAGGGACGUU	Differentiation and death of retinal progenitor cells (RPCs). Retinal development. RPE differentiation. Play an important role in the differentiation and function of RPE and retina. Increasing expression from young to adult Müller glia. Expressed in the developing retina during rod photoreceptor differentiation. Inhibition in the medaka fish results gross deficiencies in eye development. Upregulated in light adapted condition. Decreased photoreceptor apoptosis and microglia activation in mouse models of inherited retinal diseases	[60, 64–66, 90, 93, 95, 96, 100, 102–104, 107, 179]	[166]
miR-124	Adult retina, cone, rod, RPE, ONL, INL except Müller glia, GCL (adult)	ARPE-19, Mouse	> hsa-miR-124-5p MIMAT0004591 CGUGUUCACAGCGGACCUUGAU > mmu-miR-124-5p MIMAT0004527 CGUGUUCACAGCGGACCUUGAU > hsa-miR-124-3p MIMAT0000422 UAAGGCACGCGGUGAAUGCCAA > mmu-miR-124-3p MIMAT0000134 UAAGGCACGCGGUGAAUGCC	Proliferation, differentiation and death of RPCs. Connectivity and plasticity of retinal cells. Controlling the sensitivity of retinal growth cones to the guidance cue Sema3A. Regulating the survival of rod photoreceptors. Stimulating the conversion of cultured murine Müller cells into Müller glia-derived progenitor cells (MGDP). In vitro mouse Müller glia reprogramming into neural progenitors. Survival of cone photoreceptors. Exogenous supplement could be a therapeutic approach for the prevention or treatment of proliferative vitreoretinopathy. Participate in retinal cell maturation and Müller glia reprogramming. MGDP differentiation to retinal neurons. Müller glia to retinal neurons reprogramming. Decrease retinal inflammation and photoreceptor cell death and improve retinal function. Its anti-inflammatory properties have an impact as a therapeutic in treatment of retinal degenerative diseases. Promoting axon growth of RGCs differentiated from RSCs	[60, 66, 92–94, 108, 114, 143, 179–181]	[154, 163]
miR-124a	All layers except RPE, cone, all differentiated neurons, MGDP	Mouse, zebra fish	> hsa-miR-124-5p MIMAT0004591 CGUGUUCACAGCGGACCUUGAU > mmu-miR-124-5p MIMAT0004527 CGUGUUCACAGCGGACCUUGAU > dre-miR-124-5p MIMAT0031960 CGUGUUCACAGCGGACCUUGAU > hsa-miR-124-3p MIMAT0000422 UAAGGCACGCGGUGAAUGCCAA > mmu-miR-124-3p MIMAT0000134 UAAGGCACGCGGUGAAUGCC > dre-miR-124-3p MIMAT0001819 UAAGGCACGCGGUGAAUGCCAA	Controlling the maturation and survival of retinal cone photoreceptors. Expressed in all neuronal subtypes of the adult retina. Higher levels of expression in photoreceptor cells. Loss of the dominant source of miR-124a triggered death of cone photoreceptors amid retinal development. Essential for the maturation and survival of retinal cones. Knockout of one of the miR-124a genes (miR-124a-1) results in the apoptosis of newly differentiated cone photoreceptors in mice. In MGDPs committed to early neuronal lineages, upregulated during MGDP acquisition of rod phenotypes	[65, 90, 93, 99, 104, 110]	[156]
miR-181	Retina (GCL, INL), inner plexiform layer	Mouse, zebrafish	–	Retinal axon specification and growth	[90, 182]	[21, 70, 153, 160, 161]
miR-181a	Cone, amacrine cells, GCL, INL, adult retina	Mouse, zebra fish, medaka fish	> hsa-miR-181a-5p MIMAT0000256 AACAUUCAACGCUGUCGGUGAGU > mmu-miR-181a-5p MIMAT0000210 AACAUUCAACGCUGUCGGUGAGU > dre-miR-181a-5p MIMAT0001623 AACAUUCAACGCUGUCGGUGAGU > ola-miR-181a-5p MIMAT0022586 AACAUUCAACGCUGUCGGU > hsa-miR-181a-2-3p MIMAT0004558 ACCACUGACCGUUGACUGUACC > mmu-miR-181a-2-3p MIMAT0005443 ACCACCGACCGUUGACUGUACC > dre-miR-181a-2-3p MIMAT0032007 ACCAUCGACCGUUGACUGUACC > ola-miR-181a-3p MIMAT0022587 ACCAUCGACCGUUGACUGUAC	Control the assembly of visual circuitry by regulating retinal axon specification and growth. Regulate proper neuritogenesis in amacrine cells and RGCs. Expressed in amacrine cells during growth and in adult retinas. Present in both GABAergic and glycinergic amacrine cells	[60, 90, 94, 95, 99, 100, 118, 119]	[154, 158, 161]
miR-181a-5p	Retina, RPE	Human, in vitro hESC	> hsa-miR-181a-5p MIMAT0000256 AACAUUCAACGCUGUCGGUGAGU > mmu-miR-181a-5p MIMAT0000210 AACAUUCAACGCUGUCGGUGAGU > dre-miR-181a-5p MIMAT0001623 AACAUUCAACGCUGUCGGUGAGU > ola-miR-181a-5p MIMAT0022586 AACAUUCAACGCUGUCGGU	hESC differentiation into RPE cells	[124, 136]	[83]
miR-181b	Cone, amacrine cells, GCL, ciliary margin zone (CMZ), INL, mature retina	Mouse, zebra fish, medaka fish,	> hsa-miR-181b-5p MIMAT0000257 AACAUUCAUUGCUGUCGGUGGGU > mmu-miR-181b-5p MIMAT0000673 AACAUUCAUUGCUGUCGGUGGGUU > dre-miR-181b-5p MIMAT0001270 AACAUUCAUUGCUGUCGGUGGG > ola-miR-181b-5p MIMAT0022540 AACAUUCAUUGCUGUCGGUGGGUU > hsa-miR-181b-3p MIMAT0022692 CUCACUGAACAAUGAAUGCAA > mmu-miR-181b-1-3p MIMAT0017067 CUCACUGAACAAUGAAUGCAA > dre-miR-181b-3-3p MIMAT0048656 CUCACUGAACAAUGAAUGCAA > ola-miR-181b-3p MIMAT0022541 CUCACUGAACGAUGAAUGCA	Control the assembly of visual circuitry by regulating retinal axon specification and growth. Takes part in the specification of later RPCs and mature retinal neurons. Regulate proper neuritogenesis in amacrine cells and RGCs	[60, 94, 95, 99, 101, 107, 118]	[161]
miR-181c	RPE, amacrine cells, GCL, INL, MGDP	Human, mouse, zebra fish	> hsa-miR-181c-5p MIMAT0000258 AACAUUCAACCUGUCGGUGAGU > mmu-miR-181c-5p MIMAT0000674 AACAUUCAACCUGUCGGUGAGU > dre-miR-181c-5p MIMAT0001852 CACAUUCAUUGCUGUCGGUGGG > hsa-miR-181c-3p MIMAT0004559 AACCAUCGACCGUUGAGUGGAC > mmu-miR-181c-3p MIMAT0017068 ACCAUCGACCGUUGAGUGGACC > dre-miR-181c-3p MIMAT0031980 CUCGCCGGACAAUGAAUGAGAA	Promoting RPE differentiation. Upregulated during MGDP acquisition of rod phenotypes	[95, 101, 111, 129]	[158, 159, 161, 168]
miR-181d	RPE, GCL, INL	Human, mouse	> hsa-miR-181d-5p MIMAT0002821 AACAUUCAUUGUUGUCGGUGGGU > mmu-miR-181d-5p MIMAT0004324 AACAUUCAUUGUUGUCGGUGGGU > hsa-miR-181d-3p MIMAT0026608 CCACCGGGGGAUGAAUGUCAC > mmu-miR-181d-3p MIMAT0017264 CCCACCGGGGGAUGAAUGUCA	Upregulated miRNA in RPE during ESC differentiation	[101, 125]	[161]
miR-9	Müller Glia, strongly expressed in neonatal retina, CMZ maturing cells and mature amacrine cells, RPE, INL, MGDP, developing retina	ARPE-19, mouse, zebrafish	> hsa-miR-9-5p MIMAT0000441 UCUUUGGUUAUCUAGCUGUAUGA > mmu-miR-9-5p MIMAT0000142 UCUUUGGUUAUCUAGCUGUAUGA > dre-miR-9-5p MIMAT0001769 UCUUUGGUUAUCUAGCUGUAUGA > hsa-miR-9-3p MIMAT0000442 AUAAAGCUAGAUAACCGAAAGU > mmu-miR-9-3p MIMAT0000143 AUAAAGCUAGAUAACCGAAAGU > dre-miR-9-3p MIMAT0003156 UAAAGCUAGAUAACCGAAAGU	Stimulating the conversion of cultured murine Müller cells into MGDP cells. Play a significant role in orchestrating progenitor competence. Participates in the specification of later progenitor cells and mature retinal neurons. Regulate RPE cell growth, differentiation or development. Increasing expression from young to adult Müller glia. Müller glia to retinal neurons reprogramming. Rescue the effects of Dicer1 deletion on the Müller glia phenotype. Highly expressed in neonatal retina. Upregulated during MGDP acquisition of rod phenotypes (9*). Overexpression leads to decreased RPC proliferation and increased neuronal and glial differentiation. Regulate the transition between early RPCs and late RPCs. Promoted the differentiation of neuronal cells from RSCs	[66, 90, 94, 95, 99, 101, 103, 107, 108, 111, 116, 123, 183–186]	[187]
miR-182	Rod/cone/bipolar, INL (Not as vigorous as miR-183), GCL, ONL, mature retina	Mouse, Zebrafish	> hsa-miR-182-5p MIMAT0000259 UUUGGCAAUGGUAGAACUCACACU > mmu-miR-182-5p MIMAT0000211 UUUGGCAAUGGUAGAACUCACACCG > dre-miR-182-5p MIMAT0001271 UUUGGCAAUGGUAGAACUCACA > hsa-miR-182-3p MIMAT0000260 UGGUUCUAGACUUGCCAACUA > dre-miR-182-3p MIMAT0001272 UGGUUCUAGACUUGCCAACUA > mmu-miR-182-3p MIMAT0016995 GUGGUUCUAGACUUGCCAACU	May play crucial roles in the photoreceptors and bipolar cells. Maintain adult cone photoreceptor outer segments and visual function. Maintaining retinal function. Preservation of retinal nerve fiber layer thickness and preservation of RGC function. Tetramethylpyrazine protects primary RGCs against H2O2‑induced damage by suppressing apoptosis and oxidative stress via the miR‑182/mitochondrial apoptotic pathway	[90, 99, 101, 107, 120, 188, 189]	[190]
miR-183	Rod/cone/bipolar, INL (May have peripheral-to-central gradient), GCL, ONL, mature retina	Mouse, zebrafish	> hsa-miR-183-5p MIMAT0000261 UAUGGCACUGGUAGAAUUCACU > mmu-miR-183-5p MIMAT0000212 UAUGGCACUGGUAGAAUUCACU > dre-miR-183-5p MIMAT0001273 UAUGGCACUGGUAGAAUUCACUG > hsa-miR-183-3p MIMAT0004560 GUGAAUUACCGAAGGGCCAUAA > mmu-miR-183-3p MIMAT0004539 GUGAAUUACCGAAGGGCCAUAA > dre-miR-183-3p MIMAT0031921 UGAAUUACCAAAGGGCCAUAA	May play important roles in the photoreceptors and bipolar cells. Maintain adult cone photoreceptor outer segments and visual function	[99, 101, 107, 120]	[70]
miR-96	Rod/cone/bipolar, INL (Not as robust as miR-183), ONL, mature retina	Mouse, zebrafish	> hsa-miR-96-5p MIMAT0000095 UUUGGCACUAGCACAUUUUUGCU > mmu-miR-96-5p MIMAT0000541 UUUGGCACUAGCACAUUUUUGCU > dre-miR-96-5p MIMAT0001811 UUUGGCACUAGCACAUUUUUGCU > hsa-miR-96-3p MIMAT0004510 AAUCAUGUGCAGUGCCAAUAUG > mmu-miR-96-3p MIMAT0017021 CAAUCAUGUGUAGUGCCAAUAU > dre-miR-96-3p MIMAT0031956 CAAUUAUGUGUAGUGCCAAUAU	May play crucial roles in the photoreceptors and bipolar cells	[99, 101, 107]	[191]
miR-125b	CMZ, INL, GCL, developing retina	ARPE-19, in vitro hESC, mouse, zebrafish, Rat,	> hsa-miR-125b-5p MIMAT0000423 UCCCUGAGACCCUAACUUGUGA > mmu-miR-125b-5p MIMAT0000136 UCCCUGAGACCCUAACUUGUGA > rno-miR-125b-5p MIMAT0000830 UCCCUGAGACCCUAACUUGUGA > dre-miR-125b-5p MIMAT0001821 UCCCUGAGACCCUAACUUGUGA > hsa-miR-125b-2-3p MIMAT0004603 UCACAAGUCAGGCUCUUGGGAC > mmu-miR-125b-2-3p MIMAT0004529 ACAAGUCAGGUUCUUGGGACCU > rno-miR-125b-2-3p MIMAT0026467 ACAAGUCAGGCUCUUGGGACCU > dre-miR-125b-2-3p MIMAT0031964 CGGGUUGGGUUCUCGGGAGCU > hsa-miR-125b-1-3p MIMAT0004592 ACGGGUUAGGCUCUUGGGAGCU > mmu-miR-125b-1-3p MIMAT0004669 ACGGGUUAGGCUCUUGGGAGCU > rno-miR-125b-1-3p MIMAT0004730 ACGGGUUAGGCUCUUGGGAGCU > dre-miR-125b-1-3p MIMAT0031963 ACGGGUUAGGUUCUUGGGAGCU	Play a significant role in orchestrating progenitor competence. Regulate cell growth, differentiation or development. Important functions during human RPE cell differentiation	[90, 99, 107, 124, 125, 183]	[40, 154, 156, 159, 164, 165, 174]
miR-125b-5p	Retina, Müller glia	Human, in vitro hESC	> hsa-miR-125b-5p MIMAT0000423 UCCCUGAGACCCUAACUUGUGA > mmu-miR-125b-5p MIMAT0000136 UCCCUGAGACCCUAACUUGUGA	Increasing expression from young to adult Müller glia. hESC differentiation into RPE cells	[66, 124, 136]	[21, 61, 82, 83, 88, 159, 172, 173, 175]
miR-26	Rod	Mouse	> hsa-miR-26a-5p MIMAT0000082 UUCAAGUAAUCCAGGAUAGGCU > mmu-miR-26a-5p MIMAT0000533 UUCAAGUAAUCCAGGAUAGGCU > hsa-miR-26a-1-3p MIMAT0004499 CCUAUUCUUGGUUACUUGCACG > mmu-miR-26a-1-3p MIMAT0017020 CCUAUUCUUGGUUACUUGCACG > hsa-miR-26b-5p MIMAT0000083 UUCAAGUAAUUCAGGAUAGGU > mmu-miR-26b-5p MIMAT0000534 UUCAAGUAAUUCAGGAUAGGU > hsa-miR-26b-3p MIMAT0004500 CCUGUUCUCCAUUACUUGGCU > mmu-miR-26b-3p MIMAT0004630 CCUGUUCUCCAUUACUUGGCUC	Regulating the survival of rod photoreceptors	[94, 192]	[193]
miR-26a	RPE, Cone, Retina	Human, mouse	> hsa-miR-26a-5p MIMAT0000082 UUCAAGUAAUCCAGGAUAGGCU > mmu-miR-26a-5p MIMAT0000533 UUCAAGUAAUCCAGGAUAGGCU > hsa-miR-26a-2-3p MIMAT0004681 CCUAUUCUUGAUUACUUGUUUC > mmu-miR-26a-2-3p MIMAT0017058 CCUGUUCUUGAUUACUUGUUUC > hsa-miR-26a-1-3p MIMAT0004499 CCUAUUCUUGGUUACUUGCACG > mmu-miR-26a-1-3p MIMAT0017020 CCUAUUCUUGGUUACUUGCACG	Upregulated miRNA in RPE during ESC differentiation	[90, 107, 120, 125]	[154]
miR-26a-5p	Retina, RPE	Human, in vitro hESC	> hsa-miR-26a-5p MIMAT0000082 UUCAAGUAAUCCAGGAUAGGCU > mmu-miR-26a-5p MIMAT0000533 UUCAAGUAAUCCAGGAUAGGCU	hESC differentiation into RPE cells	[124, 136]	[82, 156]
miR-30	Rod	Mouse	> hsa-miR-30a-5p MIMAT0000087 UGUAAACAUCCUCGACUGGAAG > mmu-miR-30a-5p MIMAT0000128 UGUAAACAUCCUCGACUGGAAG > hsa-miR-30a-3p MIMAT0000088 CUUUCAGUCGGAUGUUUGCAGC > mmu-miR-30a-3p MIMAT0000129 CUUUCAGUCGGAUGUUUGCAGC > hsa-miR-30e-5p MIMAT0000692 UGUAAACAUCCUUGACUGGAAG > mmu-miR-30e-5p MIMAT0000248 UGUAAACAUCCUUGACUGGAAG > hsa-miR-30e-3p MIMAT0000693 CUUUCAGUCGGAUGUUUACAGC > mmu-miR-30e-3p MIMAT0000249 CUUUCAGUCGGAUGUUUACAGC > hsa-miR-30c-5p MIMAT0000244 UGUAAACAUCCUACACUCUCAGC > mmu-miR-30c-5p MIMAT0000514 UGUAAACAUCCUACACUCUCAGC > mmu-miR-30c-5p MIMAT0000514 UGUAAACAUCCUACACUCUCAGC > hsa-miR-30c-2-3p MIMAT0004550 CUGGGAGAAGGCUGUUUACUCU > mmu-miR-30c-2-3p MIMAT0005438 CUGGGAGAAGGCUGUUUACUCU > mmu-miR-30c-1-3p MIMAT0004616 CUGGGAGAGGGUUGUUUACUCC > hsa-miR-30d-5p MIMAT0000245 UGUAAACAUCCCCGACUGGAAG > mmu-miR-30d-5p MIMAT0000515 UGUAAACAUCCCCGACUGGAAG > hsa-miR-30d-3p MIMAT0004551 CUUUCAGUCAGAUGUUUGCUGC > mmu-miR-30d-3p MIMAT0017011 CUUUCAGUCAGAUGUUUGCUGC > hsa-miR-30b-5p MIMAT0000420 UGUAAACAUCCUACACUCAGCU > mmu-miR-30b-5p MIMAT0000130 UGUAAACAUCCUACACUCAGCU > hsa-miR-30b-3p MIMAT0004589 CUGGGAGGUGGAUGUUUACUUC > mmu-miR-30b-3p MIMAT0004524 CUGGGAUGUGGAUGUUUACGUC > mmu-miR-30f MIMAT0025179 GUAAACAUCCGACUGAAAGCUC	Regulating the survival of rod photoreceptors. Preservation of retinal nerve fiber layer thickness and preservation of RGC function	[94, 189, 192]	[21, 61, 70]
miR-30a	GCL, INL	Mouse	> hsa-miR-30a-5p MIMAT0000087 UGUAAACAUCCUCGACUGGAAG > mmu-miR-30a-5p MIMAT0000128 UGUAAACAUCCUCGACUGGAAG > hsa-miR-30a-3p MIMAT0000088 CUUUCAGUCGGAUGUUUGCAGC > mmu-miR-30a-3p MIMAT0000129 CUUUCAGUCGGAUGUUUGCAGC	ND	[101, 107]	[158]
miR-30b	RGC, GCL, INL, RPE	In vitro hESC, mouse, rat	> hsa-miR-30b-5p MIMAT0000420 UGUAAACAUCCUACACUCAGCU > mmu-miR-30b-5p MIMAT0000130 UGUAAACAUCCUACACUCAGCU > rno-miR-30b-5p MIMAT0000806 UGUAAACAUCCUACACUCAGCU > hsa-miR-30b-3p MIMAT0004589 CUGGGAGGUGGAUGUUUACUUC > mmu-miR-30b-3p MIMAT0004524 CUGGGAUGUGGAUGUUUACGUC > rno-miR-30b-3p MIMAT0004721 CUGGGAUGUGGAUGUUUACGUC	Upregulated in dark adaptation. Promotes axon outgrowth of RGCs. hESC differentiation into RPE cells	[90, 124, 194]	[156, 168]
miR-126	Retina	Mouse	> hsa-miR-126-5p MIMAT0000444 CAUUAUUACUUUUGGUACGCG > mmu-miR-126a-5p MIMAT0000137 CAUUAUUACUUUUGGUACGCG > hsa-miR-126-3p MIMAT0000445 UCGUACCGUGAGUAAUAAUGCG > mmu-miR-126a-3p MIMAT0000138 UCGUACCGUGAGUAAUAAUGCG	Upregulated in dark adaptation. Vascularization of the retina was severely impaired in mice that survived the miR-126 deletion. Required for the development of different retinal vascular layers. miR-126-5p is expressed in endothelial cells but also by retinal ganglion cells (RGCs) of the mouse postnatal retina and takes part in protecting endothelial cells from apoptosis during the development of the retinal vasculature. Survival of Müller cells in a mouse model using vimentin fluorescence staining. A potential therapeutic agent to keep the stability of the Blood Retina Barrier (BRB) in ischemic retinopathy. Reduces hyperglycemia-induced retinal inflammation by downregulating the HMGB1 signaling pathway	[90, 128, 195–197]	[61, 89, 152, 156, 160, 167, 168]
miR-126-3p	RPE	Human, mouse	> hsa-miR-126-3p MIMAT0000445 UCGUACCGUGAGUAAUAAUGCG > mmu-miR-126a-3p MIMAT0000138 UCGUACCGUGAGUAAUAAUGCG > mmu-miR-126b-3p MIMAT0029895 CGCGUACCAAAAGUAAUAAUGUG	Repress vascular endothelial growth factor (VEGF-A) expression in RPE cells	[101, 136, 195]	[61, 168]
miR-107	Retina	Mouse	> hsa-miR-107 MIMAT0000104 AGCAGCAUUGUACAGGGCUAUCA > mmu-miR-107-3p MIMAT0000647 AGCAGCAUUGUACAGGGCUAUCA	Upregulated in dark adaptation	[90]	[154]
miR-103	Developing retina	Mouse	> hsa-miR-103a-2-5p MIMAT0009196 AGCUUCUUUACAGUGCUGCCUUG > mmu-miR-103–2-5p MIMAT0017025 AGCUUCUUUACAGUGCUGCCUUG > hsa-miR-103a-3p MIMAT0000101 AGCAGCAUUGUACAGGGCUAUGA > mmu-miR-103-3p MIMAT0000546 AGCAGCAUUGUACAGGGCUAUGA > hsa-miR-103a-1-5p MIMAT0037306 GGCUUCUUUACAGUGCUGCCUUG > mmu-miR-103–1-5p MIMAT0017024 GGCUUCUUUACAGUGCUGCCUUG	Upregulated in dark adaptation. Regulates mitotic proliferation	[90, 107]	[154]
miR-31	MGDP cells, RPE	Mouse, zebra fish	> hsa-miR-31-5p MIMAT0000089 AGGCAAGAUGCUGGCAUAGCU > mmu-miR-31-5p MIMAT0000538 AGGCAAGAUGCUGGCAUAGCUG > hsa-miR-31-3p MIMAT0004504 UGCUAUGCCAACAUAUUGCCAU > mmu-miR-31-3p MIMAT0004634 UGCUAUGCCAACAUAUUGCCAUC > dre-miR-31 MIMAT0003347 UGGCAAGAUGUUGGCAUAGCUG	Proliferation of MGDP cells. Knockdown reduces INL proliferation at 72 h of constant light. MGDP’s proliferation	[66, 90, 101, 108, 109]	[154, 174]
Let-7	INL / GCL, rod	Mouse	–	Differentiation and death of RPCs. Regulating the survival of rod photoreceptors. Play a significant role in orchestrating progenitor competence. Participates in retinal cell maturation and Müller glia reprogramming. Influence the neuronal versus glial decision and the final differentiation of Müller glia. Critically involved in Wnt/Lin28-regulated Müller glia proliferation. May link cell proliferation to developmental time and regulate the ongoing cell cycle elongation that takes place during development. Expression maintains the differentiated state of Müller glia cells. Regulate the transition between early RPCs and late RPCs	[66, 90, 93, 94, 183, 185, 198–200]	[152]
Let-7a	RPE, retina, developing retina	Human, ARPE-19, in vitro hESC, mouse	> hsa-let-7a-5p MIMAT0000062 UGAGGUAGUAGGUUGUAUAGUU > mmu-let-7a-5p MIMAT0000521 UGAGGUAGUAGGUUGUAUAGUU > hsa-let-7a-3p MIMAT0004481 CUAUACAAUCUACUGUCUUUC > mmu-let-7a-1-3p MIMAT0004620 CUAUACAAUCUACUGUCUUUCC > hsa-let-7a-2-3p MIMAT0010195 CUGUACAGCCUCCUAGCUUUCC > mmu-let-7a-2-3p MIMAT0017015 CUGUACAGCCUCCUAGCUUUC	Upregulated miRNA in RPE during ESC Differentiation. Regulate RPE cell growth, differentiation or development. Müller glia dedifferentiation. Important functions during human RPE cell differentiation	[66, 107, 123–125]	[154, 156, 165]
Let-7a-5p	Retina	Human, in vitro hESC	> hsa-let-7a-5p MIMAT0000062 UGAGGUAGUAGGUUGUAUAGUU > mmu-let-7a-5p MIMAT0000521 UGAGGUAGUAGGUUGUAUAGUU	hESC differentiation into RPE cells	[124, 136]	[3, 82, 83, 172, 173, 175]
Let-7b	Retina, CMZ, INL, RPE, developing retina	ARPE-19, mouse, zebrafish	> hsa-let-7b-5p MIMAT0000063 UGAGGUAGUAGGUUGUGUGGUU > mmu-let-7b-5p MIMAT0000522 UGAGGUAGUAGGUUGUGUGGUU > dre-let-7b MIMAT0001760 UGAGGUAGUAGGUUGUGUGGUU > hsa-let-7b-3p MIMAT0004482 CUAUACAACCUACUGCCUUCCC > mmu-let-7b-3p MIMAT0004621 CUAUACAACCUACUGCCUUCCC	Participates in the functions of RSCs or early RPCs. Regulate RPE cell growth, differentiation or development. RPC differentiation enhancement	[90, 99, 100, 107, 123, 201]	[21, 154, 156, 158, 160, 161, 167, 168]
Let-7b-5p	RPE	Human	> hsa-let-7b-5p MIMAT0000063 UGAGGUAGUAGGUUGUGUGGUU > mmu-let-7b-5p MIMAT0000522 UGAGGUAGUAGGUUGUGUGGUU	ND	[136]	[83]
Let-7c	RPE, retina	Human, mouse	> hsa-let-7c-5p MIMAT0000064 UGAGGUAGUAGGUUGUAUGGUU > mmu-let-7c-5p MIMAT0000523 UGAGGUAGUAGGUUGUAUGGUU > hsa-let-7c-3p MIMAT0026472 CUGUACAACCUUCUAGCUUUCC > mmu-let-7c-1-3p MIMAT0004622 CUGUACAACCUUCUAGCUUUCC	Upregulated in RPE during ESC differentiation	[107, 125]	[21, 70, 154, 156, 160, 167]
Let-7c-5p	Retina	Human	> hsa-let-7c-5p MIMAT0000064 UGAGGUAGUAGGUUGUAUGGUU > mmu-let-7c-5p MIMAT0000523 UGAGGUAGUAGGUUGUAUGGUU	ND	[136]	[83]
Let-7d	INL (amacrine, bipolar), RPE, retina	ARPE-19, mouse	> hsa-let-7d-5p MIMAT0000065 AGAGGUAGUAGGUUGCAUAGUU > mmu-let-7d-5p MIMAT0000383 AGAGGUAGUAGGUUGCAUAGUU > hsa-let-7d-3p MIMAT0004484 CUAUACGACCUGCUGCCUUUCU > mmu-let-7d-3p MIMAT0000384 CUAUACGACCUGCUGCCUUUCU	Regulate RPE cell growth, differentiation or development. Plays crucial roles in neural fate specification with foreseeable function in RPC differentiation	[99, 103, 107, 123]	[154]
Let-7e	GCL, INL, photoreceptors, retina	Mouse	> hsa-let-7e-5p MIMAT0000066 UGAGGUAGGAGGUUGUAUAGUU > mmu-let-7e-5p MIMAT0000524 UGAGGUAGGAGGUUGUAUAGUU > hsa-let-7e-3p MIMAT0004485 CUAUACGGCCUCCUAGCUUUCC > mmu-let-7e-3p MIMAT0017016 CUAUACGGCCUCCUAGCUUUCC	Regulate RPE cell growth, differentiation or development. hESC differentiation into RPE cells	[101, 107, 123]	[83, 154, 156]
Let-7f	RPE, cone, developing retina	Human, Mouse	> hsa-let-7f-5p MIMAT0000067 UGAGGUAGUAGAUUGUAUAGUU > mmu-let-7f-5p MIMAT0000525 UGAGGUAGUAGAUUGUAUAGUU > hsa-let-7f-1-3p MIMAT0004486 CUAUACAAUCUAUUGCCUUCCC > mmu-let-7f-1-3p MIMAT0004623 CUAUACAAUCUAUUGCCUUCCC > hsa-let-7f-2-3p MIMAT0004487 CUAUACAGUCUACUGUCUUUCC > mmu-let-7f-2-3p MIMAT0017017 CUAUACAGUCUACUGUCUUUC	Upregulated in dark adaptation. Upregulated miRNA in RPE during ESC Differentiation	[90, 94, 107, 125]	[154, 156, 165]
Let-7f-5p	Retina, RPE	Human, in vitro hESC	> hsa-let-7f-5p MIMAT0000067 UGAGGUAGUAGAUUGUAUAGUU > mmu-let-7f-5p MIMAT0000525 UGAGGUAGUAGAUUGUAUAGUU	hESC differentiation into RPE cells	[124, 136]	[82, 88, 175]
Let-7i	RPE, retina	Human, mouse	> hsa-let-7i-5p MIMAT0000415 UGAGGUAGUAGUUUGUGCUGUU > mmu-let-7i-5p MIMAT0000122 UGAGGUAGUAGUUUGUGCUGUU > hsa-let-7i-3p MIMAT0004585 CUGCGCAAGCUACUGCCUUGCU > mmu-let-7i-3p MIMAT0004520 CUGCGCAAGCUACUGCCUUGCU	Upregulated in dark adaptation. Upregulated miRNA in RPE during ESC differentiation	[90, 107, 125]	[82, 83, 154]
miR-23a	RPE, GCL, Müller glia, retina	Human, ARPE-19, in vitro Müller glia, mouse	> hsa-miR-23a-5p MIMAT0004496 GGGGUUCCUGGGGAUGGGAUUU > mmu-miR-23a-5p MIMAT0017019 GGGGUUCCUGGGGAUGGGAUUU > hsa-miR-23a-3p MIMAT0000078 AUCACAUUGCCAGGGAUUUCC > mmu-miR-23a-3p MIMAT0000532 AUCACAUUGCCAGGGAUUUCC	Upregulated miRNA in RPE during ESC differentiation. Downregulated in the RPE derived from patients with AMD, manipulation of this miRNA modulated the susceptibility to apoptosis of RPE-derived cell lines. Increasing expression from young to adult Müller glia. Increased expression in in vitro Müller glia. Müller glia dedifferentiation. miR‐374 can work with miR‐23a to cooperatively regulate the expression of Brn3b, thereby influencing RGC development	[65, 66, 90, 101, 107, 123, 125, 202]	[154, 156, 159, 165]
miR-23a-3p	RPE, retina	Human, in vitro hESC	> hsa-miR-23a-3p MIMAT0000078 AUCACAUUGCCAGGGAUUUCC > mmu-miR-23a-3p MIMAT0000532 AUCACAUUGCCAGGGAUUUCC	hESC differentiation into RPE cells	[124, 136]	[83, 88, 173, 175, 203]
miR-106	Retina	Mouse	> hsa-miR-106a-5p MIMAT0000103 AAAAGUGCUUACAGUGCAGGUAG > mmu-miR-106a-5p MIMAT0000385 CAAAGUGCUAACAGUGCAGGUAG > hsa-miR-106a-3p MIMAT0004517 CUGCAAUGUAAGCACUUCUUAC > mmu-miR-106a-3p MIMAT0017009 ACUGCAGUGCCAGCACUUCUUAC > hsa-miR-106b-5p MIMAT0000680 UAAAGUGCUGACAGUGCAGAU > mmu-miR-106b-5p MIMAT0000386 UAAAGUGCUGACAGUGCAGAU > hsa-miR-106b-3p MIMAT0004672 CCGCACUGUGGGUACUUGCUGC > mmu-miR-106b-3p MIMAT0004582 CCGCACUGUGGGUACUUGCUGC	Key regulators of the neurogenic-to-gliogenic transition in neural progenitor cells	[66, 90]	[203]
miR-106a	GCL, INL, RPE, developing retina	Mouse	> hsa-miR-106a-5p MIMAT0000103 AAAAGUGCUUACAGUGCAGGUAG > mmu-miR-106a-5p MIMAT0000385 CAAAGUGCUAACAGUGCAGGUAG > hsa-miR-106a-3p MIMAT0004517 CUGCAAUGUAAGCACUUCUUAC > mmu-miR-106a-3p MIMAT0017009 ACUGCAGUGCCAGCACUUCUUAC	Regulates mitotic proliferation	[101, 107]	[172]
miR-143	Retina, Müller glia	In vitro Müller glia, mouse	> hsa-miR-143-5p MIMAT0004599 GGUGCAGUGCUGCAUCUCUGGU > mmu-miR-143-5p MIMAT0017006 GGUGCAGUGCUGCAUCUCUGG > hsa-miR-143-3p MIMAT0000435 UGAGAUGAAGCACUGUAGCUC > mmu-miR-143-3p MIMAT0000247 UGAGAUGAAGCACUGUAGCUC	Increased expression in in vitro Müller glia. Alleviates retinal neovascularization	[66, 90, 107, 204]	[42, 153, 154, 163]
miR-142-5p	Retina, RPE	Human	> hsa-miR-142-5p MIMAT0000433 CAUAAAGUAGAAAGCACUACU > mmu-miR-142a-5p MIMAT0000154 CAUAAAGUAGAAAGCACUACU	ND	[136]	[174]
miR-143-3p	Retina	Human	> hsa-miR-143-3p MIMAT0000435 UGAGAUGAAGCACUGUAGCUC > mmu-miR-143-3p MIMAT0000247 UGAGAUGAAGCACUGUAGCUC	ND	[136]	[82–84, 88]
miR-200b	Retina, developing retina, ganglion cell, Müller glia cell, human Müller cell line	Mouse, rat	> hsa-miR-200b-5p MIMAT0004571 CAUCUUACUGGGCAGCAUUGGA > mmu-miR-200b-5p MIMAT0004545 CAUCUUACUGGGCAGCAUUGGA > rno-miR-200b-5p MIMAT0017152 CAUCUUACUGGGCAGCAUUGGA > hsa-miR-200b-3p MIMAT0000318 UAAUACUGCCUGGUAAUGAUGA > mmu-miR-200b-3p MIMAT0000233 UAAUACUGCCUGGUAAUGAUGA > rno-miR-200b-3p MIMAT0000875 UAAUACUGCCUGGUAAUGAUGAC	The regulation of miR-200b in retinal neovascular diseases may prohibit the deviating expression of critical factors associated with pathological angiogenesis. Therapeutic effect on DR	[90, 107, 128, 134, 205]	[156]
miR-206	Retina	Human, rat	> hsa-miR-206 MIMAT0000462 UGGAAUGUAAGGAAGUGUGUGG > rno-miR-206-3p MIMAT0000879 UGGAAUGUAAGGAAGUGUGUGG	ND	[90]	[61, 166]
miR-146a	Müller glia	Human, zebra fish, rat	> hsa-miR-146a-5p MIMAT0000449 UGAGAACUGAAUUCCAUGGGUU > rno-miR-146a-5p MIMAT0000852 UGAGAACUGAAUUCCAUGGGUU > dre-miR-146a MIMAT0001843 UGAGAACUGAAUUCCAUAGAUGG > hsa-miR-146a-3p MIMAT0004608 CCUCUGAAAUUCAGUUCUUCAG > rno-miR-146a-3p MIMAT0017132 ACCUGUGAAGUUCAGUUCUUU	Proliferation of MGDP cells. Play roles in Müller glia dedifferentiation and proliferation, along with neuronal progenitor cell proliferation and migration. Its reduction reduces INL proliferation at 51 h of light treatment. The rhythmicity of miR-146a expression in the diabetic retina may proceed to mediate rhythmicity of the inflammatory response in retinal cells and provide a new approach to regulation of inflammation in DR. A potential therapeutic target for reducing inflammation in retinal microvascular endothelial cells through inhibition of TLR4/NF-*κ*B and TNF*α.* Differentiation process of human parthenogenetic embryonic stem cell (hPESC)-derived RPE cells	[91, 108, 109, 131, 206]	[21, 61, 152–156, 158–161]
miR-146a-5p	RPE	Human	> hsa-miR-146a-5p MIMAT0000449 UGAGAACUGAAUUCCAUGGGUU > mmu-miR-146a-5p MIMAT0000158 UGAGAACUGAAUUCCAUGGGUU	ND	[136]	[82]
miR-886	RPE	Human	> hsa-mir-886 MI0005527 CACUCCUACCCGGGUCGGAGUUAGCUCAAGCGGUUACCUCCUCAUGCCGGACUUUCUAUCUGUCCAUCUCUGUGCUGGGGUUCGAGACCCGCGGGUGCUUACUGACCCUUUUAUGCAAUAA	Differentiation process of hPESC-derived RPE cells	[91]	[152]
miR-10a	RPE	Human	> hsa-miR-10a-5p MIMAT0000253 UACCCUGUAGAUCCGAAUUUGUG > mmu-miR-10a-5p MIMAT0000648 UACCCUGUAGAUCCGAAUUUGUG > hsa-miR-10a-3p MIMAT0004555 CAAAUUCGUAUCUAGGGGAAUA > mmu-miR-10a-3p MIMAT0004659 CAAAUUCGUAUCUAGGGGAAUA	Differentiation process of hPESC-derived RPE cells	[91]	[193]
miR-10a-5p	RPE	Human	> hsa-miR-10a-5p MIMAT0000253 UACCCUGUAGAUCCGAAUUUGUG > mmu-miR-10a-5p MIMAT0000648 UACCCUGUAGAUCCGAAUUUGUG	ND	[136]	[82]
miR-34a	RPE, retina	ARPE-19, in vitro hESC, mouse	> hsa-miR-34a-5p MIMAT0000255 UGGCAGUGUCUUAGCUGGUUGU > mmu-miR-34a-5p MIMAT0000542 UGGCAGUGUCUUAGCUGGUUGU > hsa-miR-34a-3p MIMAT0004557 CAAUCAGCAAGUAUACUGCCCU > mmu-miR-34a-3p MIMAT0017022 AAUCAGCAAGUAUACUGCCCU	Inhibit the proliferation and migration of RPE cells. Modulated the proliferation and migration of cultured RPE cell lines. hESC differentiation into RPE cells	[65, 107, 124, 135]	[83, 156]
miR-22	Rod, RPE, Müller glia, retina	Human, in vitro Müller glia, mouse	> hsa-miR-22-5p MIMAT0004495 AGUUCUUCAGUGGCAAGCUUUA > mmu-miR-22-5p MIMAT0004629 AGUUCUUCAGUGGCAAGCUUUA > hsa-miR-22-3p MIMAT0000077 AAGCUGCCAGUUGAAGAACUGU > mmu-miR-22-3p MIMAT0000531 AAGCUGCCAGUUGAAGAACUGU	Regulating the survival of rod photoreceptors. Upregulated miRNA in RPE during ESC differentiation. Increased expression in in vitro Müller glia	[66, 94, 107, 125, 192]	[21, 40, 42, 61, 70, 154, 156, 164, 169, 170]
miR-22-3p	Retina	Human	> hsa-miR-22-3p MIMAT0000077 AAGCUGCCAGUUGAAGAACUGU > mmu-miR-22-3p MIMAT0000531 AAGCUGCCAGUUGAAGAACUGU	A suppressive task in RPE damage by targeting NLRP3, which provides novel insights into the upcoming intervention to retinopathy	[136, 207]	[82–84, 88]
miR-191	GCL, INL, ONL, cone, developing retina	Mouse	> hsa-miR-191-5p MIMAT0000440 CAACGGAAUCCCAAAAGCAGCUG > mmu-miR-191-5p MIMAT0000221 CAACGGAAUCCCAAAAGCAGCUG > hsa-miR-191-3p MIMAT0001618 GCUGCGCUUGGAUUUCGUCCCC > mmu-miR-191-3p MIMAT0004542 GCUGCACUUGGAUUUCGUUCCC	ND	[101, 107, 120]	[154, 165]
miR-191-5p	Retina	Human	> hsa-miR-191-5p MIMAT0000440 CAACGGAAUCCCAAAAGCAGCUG > mmu-miR-191-5p MIMAT0000221 CAACGGAAUCCCAAAAGCAGCUG	ND	[136]	[82, 83]
miR-127-3p	Retina	Human	> hsa-miR-127-3p MIMAT0000446 UCGGAUCCGUCUGAGCUUGGCU > mmu-miR-127-3p MIMAT0000139 UCGGAUCCGUCUGAGCUUGGCU	ND	[136]	[21, 82, 83]
miR-27b-3p	Retina, RPE	Human, in vitro hESC	> hsa-miR-27b-3p MIMAT0000419 UUCACAGUGGCUAAGUUCUGC > mmu-miR-27b-3p MIMAT0000126 UUCACAGUGGCUAAGUUCUGC	hESC differentiation into RPE cells	[124, 136]	[82, 83, 152]
miR-92	Rod, strongly expressed in neonatal retina	Mouse	> hsa-miR-92a-2-5p MIMAT0004508 GGGUGGGGAUUUGUUGCAUUAC > mmu-miR-92a-2-5p MIMAT0004635 AGGUGGGGAUUGGUGGCAUUAC > hsa-miR-92a-3p MIMAT0000092 UAUUGCACUUGUCCCGGCCUGU > mmu-miR-92a-3p MIMAT0000539 UAUUGCACUUGUCCCGGCCUG > hsa-miR-92a-1-5p MIMAT0004507 AGGUUGGGAUCGGUUGCAAUGCU > mmu-miR-92a-1-5p MIMAT0017066 AGGUUGGGAUUUGUCGCAAUGCU > hsa-miR-92b-5p MIMAT0004792 AGGGACGGGACGCGGUGCAGUG > mmu-miR-92b-5p MIMAT0017278 AGGGACGGGACGUGGUGCAGUGUU > hsa-miR-92b-3p MIMAT0003218 UAUUGCACUCGUCCCGGCCUCC > mmu-miR-92b-3p MIMAT0004899 UAUUGCACUCGUCCCGGCCUCC	Regulating the survival of rod photoreceptors. Preservation of retinal nerve fiber layer thickness and preservation of RGC function	[94, 95, 189, 192]	[12, 21, 85]
miR-92a-3p	Retina	Human, mouse	> hsa-miR-92a-3p MIMAT0000092 UAUUGCACUUGUCCCGGCCUGU > mmu-miR-92a-3p MIMAT0000539 UAUUGCACUUGUCCCGGCCUG	Retinal development	[133, 136]	[3, 82, 84, 172]
miR-92b-3p	Retina	Human	> hsa-miR-92b-3p MIMAT0003218 UAUUGCACUCGUCCCGGCCUCC > mmu-miR-92b-3p MIMAT0004899 UAUUGCACUCGUCCCGGCCUCC	Photoreceptor development and differentiation. RGC development and differentiation	[136, 208]	[82]
miR-99b	RPE, INL, photoreceptors, developing retina	Human, mouse	> hsa-miR-99b-5p MIMAT0000689 CACCCGUAGAACCGACCUUGCG > mmu-miR-99b-5p MIMAT0000132 CACCCGUAGAACCGACCUUGCG > hsa-miR-99b-3p MIMAT0004678 CAAGCUCGUGUCUGUGGGUCCG > mmu-miR-99b-3p MIMAT0004525 CAAGCUCGUGUCUGUGGGUCCG	Promoting RPE differentiation	[101, 107, 129]	[193]
miR-99b-5p	Retina	Human	> hsa-miR-99b-5p MIMAT0000689 CACCCGUAGAACCGACCUUGCG > mmu-miR-99b-5p MIMAT0000132 CACCCGUAGAACCGACCUUGCG	ND	[136]	[82]
miR-16	Retina, RPE, developing retina	ARPE-19, rabbit, mouse	> hsa-miR-16-5p MIMAT0000069 UAGCAGCACGUAAAUAUUGGCG > mmu-miR-16-5p MIMAT0000527 UAGCAGCACGUAAAUAUUGGCG > ocu-miR-16b-5p MIMAT0048107 UAGCAGCACGUAAAUAUUGGCGU > ocu-miR-16a-5p MIMAT0048105 UAGCAGCACGUAAAUACUGGCG > hsa-miR-16–1-3p MIMAT0004489 CCAGUAUUAACUGUGCUGCUGA > mmu-miR-16–1-3p MIMAT0004625 CCAGUAUUGACUGUGCUGCUGA > ocu-miR-16a-3p MIMAT0048106 CCAGUAUUAACUGUGCUGCUGAA > hsa-miR-16–2-3p MIMAT0004518 CCAAUAUUACUGUGCUGCUUUA > mmu-miR-16–2-3p MIMAT0017018 ACCAAUAUUAUUGUGCUGCUUU > ocu-miR-16b-3p MIMAT0048108 ACCAAUAUUAUUGUGCUGCUUUA	Play a role in retinal development. Regulate RPE cell growth, differentiation. Inhibition of insulin resistance in diabetic retina	[107, 123, 127, 137]	[61, 156, 165, 170, 174]
miR-16-5p	Retina, RPE	Human, in vitro hESC	> hsa-miR-16-5p MIMAT0000069 UAGCAGCACGUAAAUAUUGGCG > mmu-miR-16-5p MIMAT0000527 UAGCAGCACGUAAAUAUUGGCG	hESC differentiation into RPE cells	[124, 136]	[61, 83, 172, 173]
miR-148a	Retina	Mouse	> hsa-miR-148a-5p MIMAT0004549 AAAGUUCUGAGACACUCCGACU > mmu-miR-148a-5p MIMAT0004617 AAAGUUCUGAGACACUCCGACU > hsa-miR-148a-3p MIMAT0000243 UCAGUGCACUACAGAACUUUGU > mmu-miR-148a-3p MIMAT0000516 UCAGUGCACUACAGAACUUUGU	ND	[106, 107]	[193]
miR-148a-3p	Retina	Human	> hsa-miR-148a-3p MIMAT0000243 UCAGUGCACUACAGAACUUUGU > mmu-miR-148a-3p MIMAT0000516 UCAGUGCACUACAGAACUUUGU	Moderates high glucose-induced DR by targeting TGFB2 and FGF2	[136, 209]	[83]
miR-125a	Retina	Mouse	> hsa-miR-125a-5p MIMAT0000443 UCCCUGAGACCCUUUAACCUGUGA > mmu-miR-125a-5p MIMAT0000135 UCCCUGAGACCCUUUAACCUGUGA > hsa-miR-125a-3p MIMAT0004602 ACAGGUGAGGUUCUUGGGAGCC > mmu-miR-125a-3p MIMAT0004528 ACAGGUGAGGUUCUUGGGAGCC	Regulate the transition between early RPCs and late RPCs	[92, 125, 185]	[61, 156, 174]
miR-125a-5p	Retina, RPE, developing retina	Human, in vitro hESC, mouse	> hsa-miR-125a-5p MIMAT0000443 UCCCUGAGACCCUUUAACCUGUGA > mmu-miR-125a-5p MIMAT0000135 UCCCUGAGACCCUUUAACCUGUGA	hESC differentiation into RPE cells	[107, 124, 136]	[83, 154]
miR-100	RPE, Müller glia, developing retina	Human, mouse	> hsa-miR-100-5p MIMAT0000098 AACCCGUAGAUCCGAACUUGUG > mmu-miR-100-5p MIMAT0000655 AACCCGUAGAUCCGAACUUGUG > hsa-miR-100-3p MIMAT0004512 CAAGCUUGUAUCUAUAGGUAUG > mmu-miR-100-3p MIMAT0017051 ACAAGCUUGUGUCUAUAGGUAU	Promoting RPE differentiation. Upregulated miRNA in RPE during ESC differentiation. Increasing expression from young to adult Müller glia. Regulates mitotic proliferation	[66, 107, 125, 129]	[153, 154, 156, 159, 165, 174]
miR-100-5p	Retina	Human	> hsa-miR-100-5p MIMAT0000098 AACCCGUAGAUCCGAACUUGUG > mmu-miR-100-5p MIMAT0000655 AACCCGUAGAUCCGAACUUGUG	Upregulated during the differentiation of human embryonic stem cells into RPE Cell	[136, 210]	[82, 83, 88, 172, 173, 175]
miR-29	Neural retina, ONL	Mouse	hsa-miR-29a-5p MIMAT0004503 ACUGAUUUCUUUUGGUGUUCAG > mmu-miR-29a-5p MIMAT0004631 ACUGAUUUCUUUUGGUGUUCAG > hsa-miR-29a-3p MIMAT0000086 UAGCACCAUCUGAAAUCGGUUA > mmu-miR-29a-3p MIMAT0000535 UAGCACCAUCUGAAAUCGGUUA > hsa-miR-29b-1-5p MIMAT0004514 GCUGGUUUCAUAUGGUGGUUUAGA > mmu-miR-29b-1-5p MIMAT0004523 GCUGGUUUCAUAUGGUGGUUUA > hsa-miR-29b-3p MIMAT0000100 UAGCACCAUUUGAAAUCAGUGUU > mmu-miR-29b-3p MIMAT0000127 UAGCACCAUUUGAAAUCAGUGUU > hsa-miR-29c-5p MIMAT0004673 UGACCGAUUUCUCCUGGUGUUC > mmu-miR-29c-5p MIMAT0004632 UGACCGAUUUCUCCUGGUGUUC > hsa-miR-29c-3p MIMAT0000681 UAGCACCAUUUGAAAUCGGUUA > mmu-miR-29c-3p MIMAT0000536 UAGCACCAUUUGAAAUCGGUUA	ND	[90, 95]	[193]
miR-29a	RPCs, Müller glia, MGDP, retina	In vivo mouse RPC, in vitro Müller glia, mouse, rat	> hsa-miR-29a-5p MIMAT0004503 ACUGAUUUCUUUUGGUGUUCAG > mmu-miR-29a-5p MIMAT0004631 ACUGAUUUCUUUUGGUGUUCAG > rno-miR-29a-5p MIMAT0004718 ACUGAUUUCUUUUGGUGUUCAG > hsa-miR-29a-3p MIMAT0000086 UAGCACCAUCUGAAAUCGGUUA > mmu-miR-29a-3p MIMAT0000535 UAGCACCAUCUGAAAUCGGUUA > rno-miR-29a-3p MIMAT0000802 UAGCACCAUCUGAAAUCGGUUA	Regulates the proliferation and differentiation of RPCs. Increased expression in in vitro Müller glia. Increased in MGDPs. Protect RGCs against oxidative injury	[66, 107, 111, 146, 211]	[193]
miR-29a-3p	RPE	Human	> hsa-miR-29a-3p MIMAT0000086 UAGCACCAUCUGAAAUCGGUUA > mmu-miR-29a-3p MIMAT0000535 UAGCACCAUCUGAAAUCGGUUA	ND	[136]	[83]
miR-29b	RPE, RGC, INL, retina	ARPE-19, mouse, rat	> hsa-miR-29b-1-5p MIMAT0004514 GCUGGUUUCAUAUGGUGGUUUAGA > mmu-miR-29b-1-5p MIMAT0004523 GCUGGUUUCAUAUGGUGGUUUA > rno-miR-29b-1-5p MIMAT0005445 UUUCAUAUGGUGGUUUAGAUUU > hsa-miR-29b-3p MIMAT0000100 UAGCACCAUUUGAAAUCAGUGUU > mmu-miR-29b-3p MIMAT0000127 UAGCACCAUUUGAAAUCAGUGUU > rno-miR-29b-3p MIMAT0000801 UAGCACCAUUUGAAAUCAGUGUU	Regulates TGF-β1-mediated epithelial–mesenchymal transition of RPE cells. Protective effect against the apoptosis of RGCs and cells of the INL	[107, 139, 140]	[193]
miR-29b-3p	Retina	Human	> hsa-miR-29b-3p MIMAT0000100 UAGCACCAUUUGAAAUCAGUGUU > mmu-miR-29b-3p MIMAT0000127 UAGCACCAUUUGAAAUCAGUGUU	Inhibits cell proliferation and angiogenesis by targeting VEGF-A and PDGFB in retinal microvascular endothelial cells	[136, 212]	[83]
miR-29c	GCL, INL, photoreceptors, retina	Human, mouse, rat	> hsa-miR-29c-5p MIMAT0004673 UGACCGAUUUCUCCUGGUGUUC > mmu-miR-29c-5p MIMAT0004632 UGACCGAUUUCUCCUGGUGUUC > rno-miR-29c-5p MIMAT0003154 UGACCGAUUUCUCCUGGUGUUC > hsa-miR-29c-3p MIMAT0000681 UAGCACCAUUUGAAAUCGGUUA > mmu-miR-29c-3p MIMAT0000536 UAGCACCAUUUGAAAUCGGUUA > rno-miR-29c-3p MIMAT0000803 UAGCACCAUUUGAAAUCGGUUA	May influence neurogliogenic decision in the developing retina	[97, 101, 107, 213]	[214]
miR-151a-3p	Retina	Human	> hsa-miR-151a-3p MIMAT0000757 CUAGACUGAAGCUCCUUGAGG	ND	[136]	[82]
miR-21	Müller glia, RGC	In vitro Müller glia, in vitro Retinal microvascular endothelial cells isolated from bovine retina	> hsa-miR-21-5p MIMAT0000076 UAGCUUAUCAGACUGAUGUUGA > mmu-miR-21a-5p MIMAT0000530 UAGCUUAUCAGACUGAUGUUGA > bta-miR-21-5p MIMAT0003528 UAGCUUAUCAGACUGAUGUUGACU > hsa-miR-21-3p MIMAT0004494 CAACACCAGUCGAUGGGCUGU > mmu-miR-21a-3p MIMAT0004628 CAACAGCAGUCGAUGGGCUGUC > bta-miR-21-3p MIMAT0003745 AACAGCAGUCGAUGGGCUGUCU > mmu-miR-21b MIMAT0025121 UAGUUUAUCAGACUGAUAUUUCC > mmu-miR-21c MIMAT0025148 UAGCUUAUCAGACUGGUACAA	Increased expression in in vitro Müller glia. Pro-angiogenic role in the retinal microvasculature. Protect RGC-5 cells against oxygen glucose deprivation (OGD-induced) cells injury. Photoreceptor protection	[66, 128, 215–217]	[21, 40, 152–154, 156, 159, 160, 163–165, 174]
miR-21-5p	Retina, RPE	Human, in vitro hESC	> hsa-miR-21-5p MIMAT0000076 UAGCUUAUCAGACUGAUGUUGA > mmu-miR-21a-5p MIMAT0000530 UAGCUUAUCAGACUGAUGUUGA	hESC differentiation into RPE cells	[124, 136]	[3, 21, 61, 82–84, 88, 172, 173, 175]
miR-101-3p	RPE	Human, in vitro hESC	> hsa-miR-101-3p MIMAT0000099 UACAGUACUGUGAUAACUGAA	hESC differentiation into RPE cells	[124, 136]	[156]
miR-146b	Developing retina	Mouse	> hsa-miR-146b-5p MIMAT0002809 UGAGAACUGAAUUCCAUAGGCUG > mmu-miR-146b-5p MIMAT0003475 UGAGAACUGAAUUCCAUAGGCU > hsa-miR-146b-3p MIMAT0004766 GCCCUGUGGACUCAGUUCUGGU > mmu-miR-146b-3p MIMAT0004826 GCCCUAGGGACUCAGUUCUGGU	Regulates mitotic proliferation. Regulatory role of miR-146b-3p in diabetes related retinal inflammation by suppressing adenosine deaminase (ADA2)	[107, 218]	[21]
miR-146b-5p	RPE	Human	> hsa-miR-146b-5p MIMAT0002809 UGAGAACUGAAUUCCAUAGGCUG > mmu-miR-146b-5p MIMAT0003475 UGAGAACUGAAUUCCAUAGGCU	ND	[136]	[82]
miR-486-5p	RPE	Human	> hsa-miR-486-5p MIMAT0002177 UCCUGUACUGAGCUGCCCCGAG > mmu-miR-486a-5p MIMAT0003130 UCCUGUACUGAGCUGCCCCGAG > mmu-miR-486b-5p MIMAT0014943 UCCUGUACUGAGCUGCCCCGAG	ND	[136]	[3, 82, 84, 88]
miR-23b	RPE, retina	Human, ARPE-19, mouse	> hsa-miR-23b-5p MIMAT0004587 UGGGUUCCUGGCAUGCUGAUUU > mmu-miR-23b-5p MIMAT0016980 GGGUUCCUGGCAUGCUGAUUU > hsa-miR-23b-3p MIMAT0000418 AUCACAUUGCCAGGGAUUACCAC > mmu-miR-23b-3p MIMAT0000125 AUCACAUUGCCAGGGAUUACC	Promoting RPE differentiation. Regulate RPE cell growth, differentiation or development	[107, 123, 129]	[70, 154, 156, 164]
miR-23b-3p	RPE	Human, in vitro hESC	> hsa-miR-23b-3p MIMAT0000418 AUCACAUUGCCAGGGAUUACCAC > mmu-miR-23b-3p MIMAT0000125 AUCACAUUGCCAGGGAUUACC	hESC differentiation into RPE cells	[124, 136]	[178]
miR-145	GCL, INL, RPE, Müller glia, retinal endothelial cells	In vitro human retinal endothelial cells, in vitro Müller glia, mouse	> hsa-miR-145-5p MIMAT0000437 GUCCAGUUUUCCCAGGAAUCCCU > mmu-miR-145a-5p MIMAT0000157 GUCCAGUUUUCCCAGGAAUCCCU > mmu-miR-145b MIMAT0025105 GUCCAGUUUUCCCAGGAGACU > hsa-miR-145-3p MIMAT0004601 GGAUUCCUGGAAAUACUGUUCU > mmu-miR-145a-3p MIMAT0004534 AUUCCUGGAAAUACUGUUCUUG	Reduces high glucose-induced oxidative stress and inflammation in retinal endothelial cells. Increased expression in in vitro Müller glia. Müller glia dedifferentiation	[66, 101, 142]	[21, 154, 156, 159, 161, 164, 165]
miR-145-5p	RPE, retina	Human	> hsa-miR-145-5p MIMAT0000437 GUCCAGUUUUCCCAGGAAUCCCU > mmu-miR-145a-5p MIMAT0000157 GUCCAGUUUUCCCAGGAAUCCCU	ND	[136]	[83, 153, 172, 175]
miR-451a	RPE, retina	Human	> hsa-miR-451a MIMAT0001631 AAACCGUUACCAUUACUGAGUU > mmu-miR-451a MIMAT0001632 AAACCGUUACCAUUACUGAGUU	miR-451a/ATF2 plays a critical role in the regulation of proliferation and migration in RPE cells via regulation of mitochondrial function	[136, 219]	[83, 174]
miR-150	Retina	Mouse	> hsa-miR-150-5p MIMAT0000451 UCUCCCAACCCUUGUACCAGUG > mmu-miR-150-5p MIMAT0000160 UCUCCCAACCCUUGUACCAGUG > hsa-miR-150-3p MIMAT0004610 CUGGUACAGGCCUGGGGGACAG > mmu-miR-150-3p MIMAT0004535 CUGGUACAGGCCUGGGGGAUAG	Suppression of pathological retinal neovascularization	[151]	[154, 160]
miR-133b	Retina, amacrine cells	Rat	> hsa-miR-133b MIMAT0000770 UUUGGUCCCCUUCAACCAGCUA > mmu-miR-133b-3p MIMAT0000769 UUUGGUCCCCUUCAACCAGCUA > rno-miR-133b-3p MIMAT0003126 UUUGGUCCCCUUCAACCAGCUA > mmu-miR-133b-5p MIMAT0017083 GCUGGUCAAACGGAACCAAGUC > rno-miR-133b-5p MIMAT0017205 GCUGGUCAAACGGAACCAAGU	Differentiation and death of RPCs. Connectivity and plasticity of retinal cells. Control of the maturation and function of dopaminergic amacrine cells. Plays an important protective role in RGCs apoptosis through MAPK/Erk2 signaling pathway	[93, 220, 221]	[40, 42, 61, 156, 157, 163, 164, 166, 169, 171]
miR-196a	RPCs	*Xenopus laevis*	196a: there is no information about this Xenopus laevis miRNA in miRBase > hsa-miR-196a-5p MIMAT0000226 UAGGUAGUUUCAUGUUGUUGGG > hsa-miR-196a-1-3p MIMAT0037307 CAACAACAUUAAACCACCCGA	Proliferation, differentiation and death of RPCs	[93]	[61, 83, 174]
miR-222	RPCs, RPE	Human, *Xenopus laevis*, rabbit	> hsa-miR-222-5p MIMAT0004569 CUCAGUAGCCAGUGUAGAUCCU > mmu-miR-222-5p MIMAT0017061 CUCAGUAGCCAGUGUAGAUCC > xla-miR-222-5p MIMAT0046544 GCUCAGUAAUCAGUGUAGAUCC > hsa-miR-222-3p MIMAT0000279 AGCUACAUCUGGCUACUGGGU > mmu-miR-222-3p MIMAT0000670 AGCUACAUCUGGCUACUGGGUCU > xla-miR-222-3p MIMAT0046545 AGCUACAUCUGGCUACUGGGUCU	Differentiation and death of RPCs. Highly expressed at early developmental stages in the embryonic retina. Upregulated miRNA in RPE during ESC differentiation. Prevent the progression of retinal degeneration	[16, 93, 125, 144, 222]	[82, 83, 154, 165, 173, 174]
miR-214	RPCs, RPE, Müller glia	Human, *Xenopus laevis*, in vitro Müller glia, mouse	> hsa-miR-214-5p MIMAT0004564 UGCCUGUCUACACUUGCUGUGC > mmu-miR-214-5p MIMAT0004664 UGCCUGUCUACACUUGCUGUGC > xla-miR-214-5p MIMAT0046534 GCCUGUCUACACUUGCUGUGC > hsa-miR-214-3p MIMAT0000271 ACAGCAGGCACAGACAGGCAGU > mmu-miR-214-3p MIMAT0000661 ACAGCAGGCACAGACAGGCAGU > xla-miR-214-3p MIMAT0046535 ACAGCAGGCACAGACAGGCAG > hsa-miR-24–2-5p MIMAT0004497 UGCCUACUGAGCUGAAACACAG > mmu-miR-24–1-5p MIMAT0000218 GUGCCUACUGAGCUGAUAUCAGU > xla-miR-24a-5p MIMAT0046550 GUGCCUACUGAACUGAUAUCAGU	Differentiation and death of RPCs. Highly expressed at early developmental stages in the embryonic retina. Upregulated miRNA in RPE during ESC differentiation. Increased expression in in vitro Müller glia. May act directly to either block pathological neovascularization or prevent hyperoxia-induced vaso-obliteration	[66, 93, 125, 128, 144, 223]	[154]
miR-24	RPE, GCL,INL, retina	Human, ARPE-19, in vitro hESC, mouse, rat	> hsa-miR-24–2-5p MIMAT0004497 UGCCUACUGAGCUGAAACACAG > mmu-miR-24–2-5p MIMAT0005440 GUGCCUACUGAGCUGAAACAGU > hsa-miR-24-3p MIMAT0000080 UGGCUCAGUUCAGCAGGAACAG > mmu-miR-24-3p MIMAT0000219 UGGCUCAGUUCAGCAGGAACAG > hsa-miR-24–1-5p MIMAT0000079 UGCCUACUGAGCUGAUAUCAGU > mmu-miR-24–1-5p MIMAT0000218 GUGCCUACUGAGCUGAUAUCAGU	Promoting RPE differentiation. hESC differentiation into RPE cells. Functions as an important regulator of cell death during retinal development by repressing an apoptotic program. Preserve retina from degeneration in rats by downregulating chitinase-3-like protein 1	[101, 107, 123, 124, 129, 224, 225]	[83, 154, 172–174]
miR-24a	RPCs, RPE	*Xenopus laevis*,	> hsa-miR-24-3p MIMAT0000080 UGGCUCAGUUCAGCAGGAACAG > mmu-miR-24-3p MIMAT0000219 UGGCUCAGUUCAGCAGGAACAG > xla-miR-24a-3p MIMAT0046551 UGGCUCAGUUCAGCAGGAACAG > xla-miR-24b-3p MIMAT0011146 UGGCUCAGUUCAGCAGGAC	Repression of apoptosis in the developing neural retina. Differentiation and death of RPCs. Inhibition during development makes a reduction in eye size due to a serious increase in apoptosis in the retina, whereas overexpression is adequate to prevent apoptosis. Regulate RPE cell growth, differentiation or development. Morpholino-induced inhibition in Xenopus leads to apoptosis of RPCs	[93, 104, 145]	[193]
miR-155	RPCs, retina	Mouse, *Xenopus laevis*, zebrafish	> hsa-miR-155-5p MIMAT0000646 UUAAUGCUAAUCGUGAUAGGGGUU > mmu-miR-155-5p MIMAT0000165 UUAAUGCUAAUUGUGAUAGGGGU > dre-miR-155 MIMAT0001851 UUAAUGCUAAUCGUGAUAGGGG > hsa-miR-155-3p MIMAT0004658 CUCCUACAUAUUAGCAUUAACA > mmu-miR-155-3p MIMAT0016993 CUCCUACCUGUUAGCAUUAAC 155: there is no information about this Xenopus laevis miRNA in miRBase	Differentiation and death of RPCs. Highly expressed at early developmental stages in the embryonic retina. Potentially beneficial in retinal neovascularization therapy	[93, 99, 144, 147]	[61, 152, 158]
miR-210	Retina	Mouse	> hsa-miR-210-5p MIMAT0026475 AGCCCCUGCCCACCGCACACUG > mmu-miR-210-5p MIMAT0017052 AGCCACUGCCCACCGCACACUG > hsa-miR-210-3p MIMAT0000267 CUGUGCGUGUGACAGCGGCUGA > mmu-miR-210-3p MIMAT0000658 CUGUGCGUGUGACAGCGGCUGA	Function during retinal development	[94, 226]	[40, 156, 159]
miR-17	Retina, GCL,INL, developing retina	Mouse, rabbit	> hsa-miR-17-5p MIMAT0000070 CAAAGUGCUUACAGUGCAGGUAG > mmu-miR-17-5p MIMAT0000649 CAAAGUGCUUACAGUGCAGGUAG > ocu-miR-17-5p MIMAT0048109 CAAAGUGCUUACAGUGCAGGUAG > hsa-miR-17-3p MIMAT0000071 ACUGCAGUGAAGGCACUUGUAG > mmu-miR-17-3p MIMAT0000650 ACUGCAGUGAGGGCACUUGUAG > ocu-miR-17-3p MIMAT0048110 ACUGCAGUGAAGGCACUUGUAG	Acts in retinal development. Works as a key regulator of the neurogenic-to-gliogenic transition in neural progenitor cells. Regulates the proliferation and differentiation of RPCs. Regulates mitotic proliferation	[66, 101, 107, 127, 150]	[12, 21, 85, 156, 163, 174]
miR-410	Retina, GCL, INL	Mouse	> hsa-miR-410-5p MIMAT0026558 AGGUUGUCUGUGAUGAGUUCG > mmu-miR-410-5p MIMAT0017172 AGGUUGUCUGUGAUGAGUUCG > hsa-miR-410-3p MIMAT0002171 AAUAUAACACAGAUGGCCUGU > mmu-miR-410-3p MIMAT0001091 AAUAUAACACAGAUGGCCUGU	Efficiently downregulate VEGF-A expression. Prevent retinal angiogenesis and effectively treat Retinal Neovascularization	[101, 227]	[159, 161]
miR-27a	RPE, GCL, INL, retina	Human, in vitro hESC, mouse	> hsa-miR-27a-5p MIMAT0004501 AGGGCUUAGCUGCUUGUGAGCA > mmu-miR-27a-5p MIMAT0004633 AGGGCUUAGCUGCUUGUGAGCA > hsa-miR-27a-3p MIMAT0000084 UUCACAGUGGCUAAGUUCCGC > mmu-miR-27a-3p MIMAT0000537 UUCACAGUGGCUAAGUUCCGC	Promoting RPE differentiation. hESC differentiation into RPE cells	[101, 107, 124, 129]	[61]
miR-18a	Retina, developing retina	Human, rabbit, zebrafish, mouse	> hsa-miR-18a-5p MIMAT0000072 UAAGGUGCAUCUAGUGCAGAUAG > mmu-miR-18a-5p MIMAT0000528 UAAGGUGCAUCUAGUGCAGAUAG > ocu-miR-18a-5p MIMAT0048111 UAAGGUGCAUCUAGUGCAGAUAG > dre-miR-18a MIMAT0001779 UAAGGUGCAUCUAGUGCAGAUA > hsa-miR-18a-3p MIMAT0002891 ACUGCCCUAAGUGCUCCUUCUGG > mmu-miR-18a-3p MIMAT0004626 ACUGCCCUAAGUGCUCCUUCUG > ocu-miR-18a-3p MIMAT0048112 ACUGCCCUAAGUGCUCCUUCUGGC	Sensory perception of light. Rhodopsin-like receptor activity. Regulates NeuroD and photoreceptor differentiation in the Retina. Regulates mitotic proliferation	[107, 127, 149]	[12, 85]
miR-130b	Retina, developing retina	Rabbit, mouse	> hsa-miR-130b-5p MIMAT0004680 ACUCUUUCCCUGUUGCACUAC > mmu-miR-130b-5p MIMAT0004583 ACUCUUUCCCUGUUGCACUACU > ocu-miR-130b-5p MIMAT0048219 ACUCUUUCCCUGUUGCACUACU > hsa-miR-130b-3p MIMAT0000691 CAGUGCAAUGAUGAAAGGGCAU > mmu-miR-130b-3p MIMAT0000387 CAGUGCAAUGAUGAAAGGGCAU > ocu-miR-130b-3p MIMAT0048220 CAGUGCAAUGAUGAAAGGGCAU	Play a role in retinal development	[107, 127]	[193]
miR-20a	Retina, RPE, developing retina	In vitro hESC, mouse, rabbit	> hsa-miR-20a-5p MIMAT0000075 UAAAGUGCUUAUAGUGCAGGUAG > mmu-miR-20a-5p MIMAT0000529 UAAAGUGCUUAUAGUGCAGGUAG > ocu-miR-20a-5p MIMAT0048120 UAAAGUGCUUAUAGUGCAGGUAG > hsa-miR-20a-3p MIMAT0004493 ACUGCAUUAUGAGCACUUAAAG > mmu-miR-20a-3p MIMAT0004627 ACUGCAUUACGAGCACUUAAAG > ocu-miR-20a-3p MIMAT0048121 ACUGCAUUAUGAGCACUUAAAGU	Play a role in retinal development. hESC differentiation into RPE cells. Regulates mitotic proliferation	[107, 124, 127]	[12, 85, 163]
miR-19a	Retina, INL, GCL, RPE, developing retina	In vitro hESC, rabbit, zebrafish, mouse	> hsa-miR-19a-5p MIMAT0004490 AGUUUUGCAUAGUUGCACUACA > mmu-miR-19a-5p MIMAT0004660 UAGUUUUGCAUAGUUGCACUAC > ocu-miR-19a-5p MIMAT0048115 AGUUUUGCAUAGUUGCACUAC > dre-miR-19a-5p MIMAT0003398 CUAGUUUUGCAUAGUUGCACUA > hsa-miR-19a-3p MIMAT0000073 UGUGCAAAUCUAUGCAAAACUGA > mmu-miR-19a-3p MIMAT0000651 UGUGCAAAUCUAUGCAAAACUGA > ocu-miR-19a-3p MIMAT0048116 UGUGCAAAUCUAUGCAAAACUGA > dre-miR-19a-3p MIMAT0001782 UGUGCAAAUCUAUGCAAAACUGA	Play a role in retinal development. Regulates mitotic proliferation. hESC differentiation into RPE cells. Its intravitreal injection advances axon regeneration after optic nerve crush in adult mice, and it increases axon extension in RGCs isolated from aged human donors	[99, 107, 124, 127, 228]	[12, 21, 40, 70, 85, 156, 163, 169]
miR-93	Retina, developing retina	Rabbit, mouse	> hsa-miR-93-5p MIMAT0000093 CAAAGUGCUGUUCGUGCAGGUAG > mmu-miR-93-5p MIMAT0000540 CAAAGUGCUGUUCGUGCAGGUAG > ocu-miR-93-5p MIMAT0048176 CAAAGUGCUGUUCGUGCAGGUAG > hsa-miR-93-3p MIMAT0004509 ACUGCUGAGCUAGCACUUCCCG > mmu-miR-93-3p MIMAT0004636 ACUGCUGAGCUAGCACUUCCCG > ocu-miR-93-3p MIMAT0048177 ACUGCUGAGCUAGCACUUCCCGA	Play a role in retinal development. Regulates mitotic proliferation. Overexpression significantly diminished microglial proliferation migration and cytokine release which was associated with a decrease in loss of RGCs	[107, 127, 229]	[193]
miR-93-5p	RGC	Mouse, rat	> hsa-miR-93-5p MIMAT0000093 CAAAGUGCUGUUCGUGCAGGUAG > mmu-miR-93-5p MIMAT0000540 CAAAGUGCUGUUCGUGCAGGUAG > rno-miR-93-5p MIMAT0000817 CAAAGUGCUGUUCGUGCAGGUAG	Retinal development, (Axon guidance). Upregulation of miR-93-5p binding with PTEN suppressed the autophagy of RGCs through AKT/mTOR pathway in NMDA-induced glaucoma	[133, 230]	[83]
miR-15b	Retina, GCL, INL, RPE, developing retina	ARPE-19, mouse, rabbit	> hsa-miR-15b-5p MIMAT0000417 UAGCAGCACAUCAUGGUUUACA > mmu-miR-15b-5p MIMAT0000124 UAGCAGCACAUCAUGGUUUACA > ocu-miR-15b-5p MIMAT0048103 UAGCAGCACAUCAUGGUUUACA > hsa-miR-15b-3p MIMAT0004586 CGAAUCAUUAUUUGCUGCUCUA > mmu-miR-15b-3p MIMAT0004521 CGAAUCAUUAUUUGCUGCUCUA > ocu-miR-15b-3p MIMAT0048104 CGAAUCAUAAUUUGCUGCUCUA	Play a role in retinal development. Participates in the inhibition of insulin resistance in diabetic retina. Regulates mitotic proliferation	[101, 107, 127, 137]	[61, 83]
miR-19b	Retina, developing retina	Mouse, rabbit	> hsa-miR-19b-2-5p MIMAT0004492 AGUUUUGCAGGUUUGCAUUUCA > mmu-miR-19b-2-5p MIMAT0017010 AGUUUUGCAGAUUUGCAGUUCAGC > ocu-miR-19b-2-5p MIMAT0048119 AGUUUUGCAGGUUUGCAUUUC > hsa-miR-19b-3p MIMAT0000074 UGUGCAAAUCCAUGCAAAACUGA > mmu-miR-19b-3p MIMAT0000513 UGUGCAAAUCCAUGCAAAACUGA > ocu-miR-19b-3p MIMAT0048118 UGUGCAAAUCCAUGCAAAACUGA > hsa-miR-19b-1-5p MIMAT0004491 AGUUUUGCAGGUUUGCAUCCAGC > mmu-miR-19b-1-5p MIMAT0017065 AGUUUUGCAGGUUUGCAUCCAGC > ocu-miR-19b-5p MIMAT0048117 AGUUUUGCAGGUUUGCAUCCAGC	Play a role in retinal development. Regulates mitotic proliferation	[107, 127]	[12, 85, 163, 174]
miR-19b-3p	RPE	In vitro hESC	> hsa-miR-19b-3p MIMAT0000074 UGUGCAAAUCCAUGCAAAACUGA > mmu-miR-19b-3p MIMAT0000513 UGUGCAAAUCCAUGCAAAACUGA	hESC differentiation into RPE cells	[124]	[83, 172]
miR-151b	RPE	Human	> hsa-miR-151b MIMAT0010214 UCGAGGAGCUCACAGUCU	Upregulated in RPE during ESC differentiation	[125]	[231]
miR-25	MGDP cells, developing retina	Mouse	> hsa-miR-25-5p MIMAT0004498 AGGCGGAGACUUGGGCAAUUG > mmu-miR-25-5p MIMAT0017049 AGGCGGAGACUUGGGCAAUUGC > hsa-miR-25-3p MIMAT0000081 CAUUGCACUUGUCUCGGUCUGA > mmu-miR-25-3p MIMAT0000652 CAUUGCACUUGUCUCGGUCUGA	Reprogram mouse Müller glia into neural progenitors in vitro. Regulates mitotic proliferation	[107, 108]	[83, 172]
miR-132	RGC, CMZ, INL, GCL, RPE, retina	Mouse, zebrafish	> hsa-miR-132-5p MIMAT0004594 ACCGUGGCUUUCGAUUGUUACU > mmu-miR-132-5p MIMAT0016984 AACCGUGGCUUUCGAUUGUUAC > dre-miR-132-5p MIMAT0003403 ACCGUGGCAUUAGAUUGUUACU > hsa-miR-132-3p MIMAT0000426 UAACAGUCUACAGCCAUGGUCG > mmu-miR-132-3p MIMAT0000144 UAACAGUCUACAGCCAUGGUCG > dre-miR-132-3p MIMAT0001829 UAACAGUCUACAGCCAUGGUCG	Branching of RGC axons	[65, 99, 101, 107, 232]	[154, 156, 160, 167]
miR-449	RPE	Zebrafish	449: there is no information about this zebrafish miRNA in miRBase > hsa-miR-449a MIMAT0001541 UGGCAGUGUAUUGUUAGCUGGU > hsa-miR-449c-5p MIMAT0010251 UAGGCAGUGUAUUGCUAGCGGCUGU > hsa-miR-449c-3p MIMAT0013771 UUGCUAGUUGCACUCCUCUCUGU > hsa-miR-449b-5p MIMAT0003327 AGGCAGUGUAUUGUUAGCUGGC > hsa-miR-449b-3p MIMAT0009203 CAGCCACAACUACCCUGCCACU	Consistently upregulated along with the RPE differentiation	[126]	[174]
miR-361	Retina	Human	> hsa-miR-361-5p MIMAT0000703 UUAUCAGAAUCUCCAGGGGUAC > mmu-miR-361-5p MIMAT0000704 UUAUCAGAAUCUCCAGGGGUAC > hsa-miR-361-3p MIMAT0004682 UCCCCCAGGUGUGAUUCUGAUUU > mmu-miR-361-3p MIMAT0017075 UCCCCCAGGUGUGAUUCUGAUUUGU	Overexpression of miR-361-5p might act as a suppressor in retinoblastoma. miR-361-3p functions as a tumor suppressor in the carcinogenesis and progression of retinoblastoma	[97, 233, 234]	[154]
miR-130a	GCL, INL, RPE, developing retina	Mouse	> hsa-miR-130a-5p MIMAT0004593 GCUCUUUUCACAUUGUGCUACU > mmu-miR-130a-5p MIMAT0016983 GCUCUUUUCACAUUGUGCUACU > hsa-miR-130a-3p MIMAT0000425 CAGUGCAAUGUUAAAAGGGCAU > mmu-miR-130a-3p MIMAT0000141 CAGUGCAAUGUUAAAAGGGCAU	Regulates mitotic proliferation	[101, 107]	[156, 160, 167]
miR-130a-3p	Retina	Mouse	> hsa-miR-130a-3p MIMAT0000425 CAGUGCAAUGUUAAAAGGGCAU > mmu-miR-130a-3p MIMAT0000141 CAGUGCAAUGUUAAAAGGGCAU	Retinal development	[133]	[83]
miR-320	RPE, developing retina	ARPE-19, mouse	> hsa-miR-320a-5p MIMAT0037311 GCCUUCUCUUCCCGGUUCUUCC > mmu-miR-320-5p MIMAT0017057 GCCUUCUCUUCCCGGUUCUUCC > hsa-miR-320a-3p MIMAT0000510 AAAAGCUGGGUUGAGAGGGCGA > mmu-miR-320-3p MIMAT0000666 AAAAGCUGGGUUGAGAGGGCGA > hsa-miR-320b MIMAT0005792 AAAAGCUGGGUUGAGAGGGCAA > hsa-miR-320d MIMAT0006764 AAAAGCUGGGUUGAGAGGA > hsa-miR-320e MIMAT0015072 AAAGCUGGGUUGAGAAGG > hsa-miR-320c MIMAT0005793 AAAAGCUGGGUUGAGAGGGU	Regulate RPE cell growth, differentiation or development	[107, 123]	[3, 83, 154]
miR-149	GCL, INL, RPE	Mouse	> hsa-miR-149-5p MIMAT0000450 UCUGGCUCCGUGUCUUCACUCCC > mmu-miR-149-5p MIMAT0000159 UCUGGCUCCGUGUCUUCACUCCC > hsa-miR-149-3p MIMAT0004609 AGGGAGGGACGGGGGCUGUGC > mmu-miR-149-3p MIMAT0016990 GAGGGAGGGACGGGGGCGGUGC	ND	[101]	[154]
miR-296-5p	GCL, INL, RPE	Mouse	> hsa-miR-296-5p MIMAT0000690 AGGGCCCCCCCUCAAUCCUGU > mmu-miR-296-5p MIMAT0000374 AGGGCCCCCCCUCAAUCCUGU	ND	[101]	[154]
miR-328	GCL, INL, RPE	Mouse	> hsa-miR-328-5p MIMAT0026486 GGGGGGGCAGGAGGGGCUCAGGG > mmu-miR-328-5p MIMAT0017030 GGGGGGCAGGAGGGGCUCAGGG > hsa-miR-328-3p MIMAT0000752 CUGGCCCUCUCUGCCCUUCCGU > mmu-miR-328-3p MIMAT0000565 CUGGCCCUCUCUGCCCUUCCGU	Promotion of RPE proliferation	[101, 235]	[166]
miR-294	GCL, INL, RPE	Mouse	> mmu-miR-294-5p MIMAT0004574 ACUCAAAAUGGAGGCCCUAUCU > mmu-miR-294-3p MIMAT0000372 AAAGUGCUUCCCUUUUGUGUGU 294: there is no information about this human miRNA in miRBase	May keep Müller cells pluripotency	[101, 236]	[156]
miR-221	GCL, INL	Mouse	> hsa-miR-221-5p MIMAT0004568 ACCUGGCAUACAAUGUAGAUUU > mmu-miR-221-5p MIMAT0017060 ACCUGGCAUACAAUGUAGAUUUCUGU > hsa-miR-221-3p MIMAT0000278 AGCUACAUUGUCUGCUGGGUUUC > mmu-miR-221-3p MIMAT0000669 AGCUACAUUGUCUGCUGGGUUUC	ND	[101]	[3, 40, 83, 153, 154, 156, 157, 165, 173, 174]
miR-15a	GCL, developing retina	Mouse	> hsa-miR-15a-5p MIMAT0000068 UAGCAGCACAUAAUGGUUUGUG > mmu-miR-15a-5p MIMAT0000526 UAGCAGCACAUAAUGGUUUGUG > hsa-miR-15a-3p MIMAT0004488 CAGGCCAUAUUGUGCUGCCUCA > mmu-miR-15a-3p MIMAT0004624 CAGGCCAUACUGUGCUGCCUCA	Anti-inflammatory and anti-angiogenic action of miR-15a in DR	[101, 107, 237]	[61]
miR-15a-5p	RPE	In vitro hESC	> hsa-miR-15a-5p MIMAT0000068 UAGCAGCACAUAAUGGUUUGUG > mmu-miR-15a-5p MIMAT0000526 UAGCAGCACAUAAUGGUUUGUG	hESC differentiation into RPE cells	[124]	[83]
miR-223	GCL, INL	Mouse, zebrafish	> hsa-miR-223-5p MIMAT0004570 CGUGUAUUUGACAAGCUGAGUU > mmu-miR-223-5p MIMAT0017056 CGUGUAUUUGACAAGCUGAGUUG > hsa-miR-223-3p MIMAT0000280 UGUCAGUUUGUCAAAUACCCCA > mmu-miR-223-3p MIMAT0000665 UGUCAGUUUGUCAAAUACCCCA > dre-miR-223 MIMAT0001290 UGUCAGUUUGUCAAAUACCCC	Necessary for maintaining normal retinal function as well as regulating inflammation in microglia and macrophages. Key role in zebrafish optic nerve regeneration. Upregulation of miR-223 in RGCs via intravitreal injection protected RGC axons in the optic nerve from degeneration	[101, 238–241]	[21, 61, 70, 156, 158, 174]
miR-497	GCL, INL	Mouse	> hsa-miR-497-5p MIMAT0002820 CAGCAGCACACUGUGGUUUGU > mmu-miR-497a-5p MIMAT0003453 CAGCAGCACACUGUGGUUUGUA > mmu-miR-497b MIMAT0031404 CACCACAGUGUGGUUUGGACGUGG > hsa-miR-497-3p MIMAT0004768 CAAACCACACUGUGGUGUUAGA > mmu-miR-497a-3p MIMAT0017247 CAAACCACACUGUGGUGUUAG	Functions as a tumor suppressor in the carcinogenesis and progression of retinoblastoma via targeting VEGF-A. Metformin may obstruct the VEGF-A protein translation via inducing a VEGF-A-targeting microRNA, microRNA-497a-5p, resulting in reduced retina neovascularization	[101, 242, 243]	[176]
miR-28	Retina	Mouse	> hsa-miR-28-5p MIMAT0000085 AAGGAGCUCACAGUCUAUUGAG > mmu-miR-28a-5p MIMAT0000653 AAGGAGCUCACAGUCUAUUGAG > mmu-miR-28c MIMAT0019339 AGGAGCUCACAGUCUAUUGA > mmu-miR-28b MIMAT0019354 AGGAGCUCACAAUCUAUUUAG > hsa-miR-28-3p MIMAT0004502 CACUAGAUUGUGAGCUCCUGGA > mmu-miR-28a-3p MIMAT0004661 CACUAGAUUGUGAGCUGCUGGA	Inhibits differentiation of MGDPs toward a photoreceptor lineage fate. Potentially regulates the photoreceptor lineage commitment of MGDPs	[60, 141]	[82]
miR-99a	Müller glia	Mouse	> hsa-miR-99a-5p MIMAT0000097 AACCCGUAGAUCCGAUCUUGUG > mmu-miR-99a-5p MIMAT0000131 AACCCGUAGAUCCGAUCUUGUG > hsa-miR-99a-3p MIMAT0004511 CAAGCUCGCUUCUAUGGGUCUG > mmu-miR-99a-3p MIMAT0016981 CAAGCUCGUUUCUAUGGGUCU	Increasing expression from young to adult Müller glia	[66]	[174]
miR-199a	Müller glia	In vitro Müller glia	> hsa-miR-199a-5p MIMAT0000231 CCCAGUGUUCAGACUACCUGUUC > mmu-miR-199a-5p MIMAT0000229 CCCAGUGUUCAGACUACCUGUUC > hsa-miR-199a-3p MIMAT0000232 ACAGUAGUCUGCACAUUGGUUA > mmu-miR-199a-3p MIMAT0000230 ACAGUAGUCUGCACAUUGGUUA	Increased expression in in vitro Müller glia	[66]	[61, 83, 154, 156, 161, 165, 175]
miR-140	Retina	Mouse	> hsa-miR-140-5p MIMAT0000431 CAGUGGUUUUACCCUAUGGUAG > mmu-miR-140-5p MIMAT0000151 CAGUGGUUUUACCCUAUGGUAG > hsa-miR-140-3p MIMAT0004597 UACCACAGGGUAGAACCACGG > mmu-miR-140-3p MIMAT0000152 UACCACAGGGUAGAACCACGG	MiR-140-5p suppresses retinoblastoma cell growth by inhibiting c-Met/AKT/mTOR pathway. Intravitreal delivery offers protection in preventing oxidative stress mediated retinal ischemia–reperfusion injury	[106, 107, 244, 245]	[162]
miR-151-5p	Retina	Mouse	> hsa-miR-151a-5p MIMAT0004697 UCGAGGAGCUCACAGUCUAGU > mmu-miR-151-5p MIMAT0004536 UCGAGGAGCUCACAGUCUAGU	ND	[107]	[154, 156]
miR-195	Mature retina	Mouse	> hsa-miR-195-5p MIMAT0000461 UAGCAGCACAGAAAUAUUGGC > mmu-miR-195a-5p MIMAT0000225 UAGCAGCACAGAAAUAUUGGC > hsa-miR-195-3p MIMAT0004615 CCAAUAUUGGCUGUGCUGCUCC > mmu-miR-195a-3p MIMAT0017000 CCAAUAUUGGCUGUGCUGCUCC > mmu-miR-195b MIMAT0025076 UAGCAGCACAGAAAUAGUAGAA	ND	[107]	[83, 165]
miR-423-5p	Developing retina	Mouse	> hsa-miR-423-5p MIMAT0004748 UGAGGGGCAGAGAGCGAGACUUU > mmu-miR-423-5p MIMAT0004825 UGAGGGGCAGAGAGCGAGACUUU	ND	[107]	[3, 82]
miR-374	Developing retina	Mouse	> hsa-miR-374a-5p MIMAT0000727 UUAUAAUACAACCUGAUAAGUG > hsa-miR-374a-3p MIMAT0004688 CUUAUCAGAUUGUAUUGUAAUU > hsa-miR-374b-5p MIMAT0004955 AUAUAAUACAACCUGCUAAGUG > mmu-miR-374b-5p MIMAT0003727 AUAUAAUACAACCUGCUAAGUG > hsa-miR-374b-3p MIMAT0004956 CUUAGCAGGUUGUAUUAUCAUU > mmu-miR-374b-3p MIMAT0003728 GGUUGUAUUAUCAUUGUCCGAG > hsa-miR-374c-5p MIMAT0018443 AUAAUACAACCUGCUAAGUGCU > mmu-miR-374c-5p MIMAT0014953 AUAAUACAACCUGCUAAGUG > hsa-miR-374c-3p MIMAT0022735 CACUUAGCAGGUUGUAUUAUAU > mmu-miR-374c-3p MIMAT0014954 ACUUAGCAGGUUGUAUUAU	miR‐374 can work with miR‐23a to cooperatively regulate the expression of Brn3b, thereby influencing RGC development. miR‐374a is a negative regulator of Fas death receptor which is able to enhance the cell survival and protect RPE cells against oxidative conditions	[107, 202, 246, 247]	[83]

ILM, inner limiting membrane; GCL, ganglion cell layer; IPL, inner plexiform layer; INL, inner nuclear layer; OPL, outer plexiform layer; ONL, outer nuclear layer; IS, inner segment of photoreceptors; OS, outer segment of photoreceptors; RPE, retinal pigment epithelium; MGDP, Müller glia-derived progenitor cells; CMZ, ciliary margin zone; RPC, retinal progenitor cells; RSC, retinal stem cells; ESC, embryonic stem cells; hESC, human embryonic stem cells; hPESC, human parthenogenetic embryonic stem cells; AMD, age-related macular degeneration; DR, diabetic retinopathy; ND, not defined. Human: *Homo sapiens* (hsa); Medaka fish: *Oryzias latipes* (ola); Mouse: *Mus musculus* (mmu); Rabbit: *Oryctolagus cuniculus* (ocu); Rat*: Rattus norvegicus* (rno); *Xenopus laevis* (xla); Zebrafish: *Danio rerio* (dre). All miRNA sequences are taken from www.mirbase.org

## Conclusions

miRNAs have complicated functions in retinal health and disease which most of them are yet to be understood. Each miRNA can regulate the whole genetic program of a cell, so knowing their specific effects on different types of cells could be helpful for designing more beneficent studies and therapies. Owing to the fact that a miRNA has many mRNA targets, we should consider that we still don’t know many functions of miRNAs and the procedures of their actions. Although multifunctional miRNAs such as miR-204, miR-124 seem more promising, the timing of their application should be planned more precisely to avoid undesired effects. Besides having other therapeutic agents, MSC-EVs are a great source of miRNAs which make them a good choice for a multifactorial therapy.

Identifying miRNAs that are common between retinal cells and MSC-EVs, with due attention to the role of miRNAs as master regulators, could help us to preserve or restore the state of retinal cells in a more accurate way in retinal degenerative diseases.

## Data Availability

Not applicable.

## Abbreviations

Ago: Argonaute
Ago2: Argonaute2
AMD: Age-related macular degeneration
ARPE-19: A human retinal pigment epithelial cell line
BMSC: Bone marrow mesenchymal stem cells
BRB: Blood retina barrier
CMZ: Ciliary margin zone
CNS: Central nervous system
DR: Diabetic retinopathy
ESC: Embryonic stem cells
EV: Extracellular vesicles
GCL: Ganglionic cell layer
hBMSC: Human bone marrow mesenchymal stem cells
hESC: Human embryonic stem cells
hnRNP: Heterogeneous nuclear ribonucleoproteins
hPESC: Human parthenogenetic embryonic stem cell
hRPE: Human retinal pigment epithelium
IBD: Inflammatory bowel disease
INL: Inner nuclear layer
IPF: Idiopathic pulmonary fibrosis
IPL: Inner plexiform layer
iPSCs: Induced pluripotent stem cells
ISCT: International Society for Cellular Therapy
MG: Müller glia
MGDP: Müller glia-derived progenitor cells
miRNA: MicroRNA
mRNA: Messenger RNA
MSCs: Mesenchymal stem cells
MSC-EVs: Mesenchymal stem cells extracellular vesicles
MSC-Exos: Mesenchymal stem cells exosomes
MVB: Multivesicular bodies
ONL: Outer nuclear layer
OS: Outer segments
PTEN: Phosphatase and tensin homolog
RBVS: Retinoblastoma vitreous seeding
RISC: RNA-induced silencing complex
RPC: Retinal progenitor cells
RSC: Retinal stem cells
RPE: Retinal pigment epithelium
siRNA: Short interfering RNA
SYNCRIP: Synaptotagmin-binding cytoplasmic RNA-interacting protein
VEGF-A: : Vascular endothelial growth factor
WJ-MSC: Wharton’s jelly mesenchymal stem cell
